# Multivariate Analysis of Root Architecture, Morpho-Physiological, and Biochemical Traits Reveals Higher Nitrogen Use Efficiency Heterosis in Maize Hybrids During Early Vegetative Growth

**DOI:** 10.3390/plants14030399

**Published:** 2025-01-29

**Authors:** Muhammad Faheem Jan, Ming Li, Changzhuang Liu, Waqas Liaqat, Muhammad Tanveer Altaf, Celaleddin Barutçular, Faheem Shehzad Baloch

**Affiliations:** 1 College of Agriculture, Northeast Agricultural University, Harbin 150030, China; mfaheemjan366@gmail.com (M.F.J.); liu891578576@163.com (C.L.); 2 Department of Field Crops, Faculty of Agriculture, Institute of Natural and Applied Sciences, Çukurova University, Adana 01330, Türkiye; waqasliaqat789@gmail.com (W.L.); cbarutcular@gmail.com (C.B.); 3 Department of Field Crops, Faculty of Agriculture, Recep Tayyip Erdoğan University, Pazar, Rize 53300, Türkiye; 4 Department of Biotechnology, Faculty of Science, Mersin University, Mersin 33343, Türkiye; balochfaheem13@gmail.com; 5 Department of Plant Resources and Environment, Jeju National University, Jeju 63243, Republic of Korea

**Keywords:** chlorophyll fluorescence, heterosis, nitrate reductase, nitrogen accumulation, root activity, soluble protein

## Abstract

Maize (*Zea mays* L.) is a globally significant crop with high economic and nutritional importance. Its productivity, however, relies heavily on nitrogen (N) inputs, often resulting in low nitrogen use efficiency (NUE). Enhancing NUE necessitates a comprehensive understanding of the biochemical and physiological mechanisms driving N uptake and utilization. The study evaluated the NUE heterosis of 7 inbred lines and their 12 hybrids under low and high N conditions during early vegetative growth. Significant genotypic variations across traits were analyzed using analysis of variance, principal component analysis, correlation, regression, and structural equation modeling. The key contributors to genetic variation included shoot dry weight, N accumulation, and NUE. Hybrids demonstrated enhanced root architecture, superior enzymatic activities of nitrate reductase (NR) and glutamine synthetase (GS), and improved morphological traits, photosynthetic efficiency, and N accumulation, resulting in greater biomass production, N accumulation, and NUE compared to inbred lines. Among hybrids, Zheng58 × PH4CV exhibited the highest NUE, driven by efficient N uptake, robust enzymatic activity, and substantial N accumulation. Nitrogen uptake efficiency (NUpE) correlated strongly with root traits such as activity (r = 0.80 ***), length (r = 0.73 ***), surface area (r = 0.67 ***), GS activity (r = 0.84 ***), and dry weight (r = 0.92). Similarly, nitrogen utilization efficiency (NutE) was positively correlated with shoot NR activity (r = 0.90 ***), shoot GS activity (r = 0.56 ***), leaf area (r = 0.73 ***), shoot dry weight (r = 0.82 ***), and shoot N accumulation (r = 0.55 ***), particularly under high N conditions. Based on key traits such as shoot dry weight, N accumulation, and NUE, hybrids Zheng58 × PH4CV, 444 × PH4CV, 444 × MO17, and B73 × MO17 emerged as N-efficient genotypes, confirmed by contrasting root systems, enhanced N metabolism, and superior NUE. These findings reveal the pivotal roles of root architecture and N metabolism in optimizing NUE, emphasizing the biochemical and physiological traits crucial for developing highly N-efficient maize hybrids.

## 1. Introduction

Maize (*Zea mays* L.) ranks among the most significant staple crops worldwide, contributing to nearly 38% of the total global cereal production in 2022 [1]. In recent decades, its production has surged due to rising demand, technological advancements, yield improvements, and expanded cultivation, making it the leading cereal by production volume and poised to become the most widely grown and traded crop in the next decade [2]. In China, maize cultivation covered the largest area of the total cereals harvest area, accounting for 43.1 million hectares out of a total area (99.54 million hectares), and yielded 277.21 million tons of the total cereals production (635.09 million tons) in 2022 [1]. Over the past two decades, improvements in global crop yields have predominantly been driven by increases in yield per unit area rather than the expansion of arable land [3]. The planting area of maize in Northeast China accounts for 30.6% of the whole country, which plays an important role in the food production chain of China, with Heilongjiang Province, as the major producer, reaching 15.6% of the national annual production [4,5]. According to FAOSTAT [6], the leading global producer of maize is the USA, with a production of 348.75 million tons, followed by China (277.21 million tons), Brazil (109.42 million tons), and Argentina (59.03 million tons).

Nitrogen is a critical and limiting factor for plant growth and productivity [7,8], essential for various macromolecules, metabolites, and signaling compounds that are crucial to plant development [9,10]. Maize requires substantial N input to sustain higher yield potentials [2,11]. However, inadequate N availability can reduce yields [12,13], leading to heavy reliance on nitrogenous fertilizers for growth enhancement [14,15]. Excessive use of these fertilizers decreases nitrogen use efficiency (NUE), leads to environmental problems like groundwater pollution and soil degradation [16,17], and raises production costs [18]. To tackle these challenges, identifying and developing maize genotypes with improved NUE are crucial [11,19].

The concept of NUE is multifaceted, encompassing nitrogen uptake efficiency (NUpE), which reflects the plant’s ability to absorb N from the soil, and nitrogen utilization efficiency (NUtE), which indicates how effectively the absorbed N is converted into biomass [20,21]. Genetic variation in maize genotypes, such as root morphology [22], N assimilation enzyme activities [23], chlorophyll content, photosynthetic efficiency [24], and N accumulation [25], play crucial roles in NUE optimization [26,27]. Genotypes with maximum root length (RL), root surface area (RSA), root volume (RV), diameter, root activity, root dry weight, and NUpE are strongly associated with NUE, highlighting root characteristics as key targets for enhancing biomass under both high and limited N inputs [28,29,30].

Root architecture, characterized by root length, surface area, diameter, and volume, is fundamental to N uptake and thus impacts NUE directly. The root system’s ability to explore a greater soil volume allows for better nutrient and water acquisition, which is especially crucial under N-limited conditions [31,32]. Genotypes with an enhanced root system structure are more efficient in N uptake, especially under low N conditions, due to their ability to sustain nutrient absorption across developmental stages [33,34]. Studies exploring heterosis in maize showed that hybrids exhibited significant heterosis in root traits, such as surface area and volume, contributing to higher NUpE than their inbred lines. This heterotic advantage is particularly beneficial in nutrient-limited environments where efficient nutrient scavenging is critical. Therefore, understanding and selecting root traits that promote N uptake in maize can improve NUE [35].

The enzymes nitrate reductase (NR) and glutamine synthetase (GS) are central to N metabolism, as they catalyze critical steps in N assimilation. NR and GS activities have been linked to increased NUpE and NutE, with higher enzymatic activities supporting better N conversion and remobilization, which are crucial for enhancing NUE [26,36]. Maize genotypes with high NR and GS activities exhibited improved NUE through efficient N assimilation and redistribution [37]. Hybrids displayed heterotic effects for NR and GS activities, resulting in superior N assimilation capacity compared to their inbred lines. Heterosis for NR and GS activities correlated with increased biomass production, especially under high N conditions, indicating that these enzymatic traits can be advantageous in hybrids for higher NUE [38]. These studies suggested that genotypes with higher NR and GS activities can facilitate more effective N assimilation, which is crucial for maintaining productivity under both high and low N inputs.

Photosynthetic efficiency is another critical factor influencing NUE, as it determines how effectively the plant converts absorbed N into biomass. Chlorophyll content directly correlates with photosynthetic capacity and, therefore, with N utilization. Hybrids with higher chlorophyll content maintain superior photosynthetic rates, contributing to increased biomass accumulation and improved NUE [39,40]. Additionally, N application after silking can sustain chlorophyll levels, minimizing the negative effects of N deficiency on photosynthesis and thus supporting NUE [41]. The role of chlorophyll fluorescence (CF) parameters, such as Fv/Fm (maximum photochemical efficiency of photosystem II) and Y (II) (quantum yield of PSII), are also significant. Higher Fv/Fm and Y (II) values indicate efficient energy transfer within the photosynthetic apparatus, enhancing photosynthetic resilience under variable N conditions [42]. Hybrids exhibiting superior CF parameters had better NUE due to their ability to maintain photosynthetic efficiency across N treatments [40,43]. Therefore, chlorophyll content and photosynthetic efficiency indicators are valuable traits for improving NUE, especially under N-limited conditions where maximizing photosynthetic performance is essential.

Biomass accumulation is a key outcome of efficient N utilization, as it reflects the plant’s capacity to convert absorbed N into structural and reproductive growth. Under high N conditions, hybrids often exhibit higher biomass accumulation than their inbred lines. NUE is closely related to biomass yield, particularly under conditions where N availability aligns with plant growth demands [44]. Efficient N partitioning, particularly an optimized allocation of N to roots and shoots, supports sustained growth and high biomass in maize under varied N conditions [3]. Furthermore, the heterotic response in biomass accumulation among maize hybrids was strongly associated with their enhanced ability to absorb and utilize N, allowing for more productive growth under low and high N supply [39]. The relationships among heterosis, biomass production, and NUE suggest that selecting biomass-related traits can contribute significantly to NUE improvement.

Heterosis, or hybrid vigor, plays a substantial role in the NUE of maize hybrids. NUE heterosis in maize hybrids was driven by higher N metabolism enzyme activity, improved N uptake, and internal N utilization efficiency, resulting in greater biomass production than inbred lines [45]. Several studies have reported that genetic and environmental factors influence NUE and that heterosis is a mechanism for leveraging genetic diversity to improve NUE [46,47,48]. These studies showed that hybrids with higher NUE were characterized by efficient N assimilation and utilization pathways, allowing reduced N input without compromising biomass. Based on these insights, the present study will examine the relationships among root growth, N metabolism enzyme activity, N accumulation, and NUE in maize inbred lines and their hybrids.

## 2. Results

### 2.1. Genotypic Variation in Root Activity

Root activity (RA), a key indicator of root health and nutrient absorption, is significantly influenced by genotype, nitrogen availability, and their interaction. At the V5 and V7 growth stages, significant differences in RA were observed between inbred lines and hybrids. In the absence of nitrogen application at V5, RA was lower across all genotypes, reflecting their inherent genetic potential. By contrast, nitrogen supply at V7 significantly increased RA, with the highest activity recorded at 0.46 g N/plant. Hybrids generally exhibited a more pronounced increase in RA than inbred lines, demonstrating their superior nitrogen uptake capacity. The highest RA values were observed at both stages in hybrids Zheng 58 × PH4CV, B73 × MO17, and 444 × MO17, while inbred lines MO17, PH4CV, PH6WC, and B73 demonstrated relatively higher root activity (Supplementary Figure S1).

### 2.2. Genotypic Variation in Root Characteristics at V5 Stage

**Figure 1:**
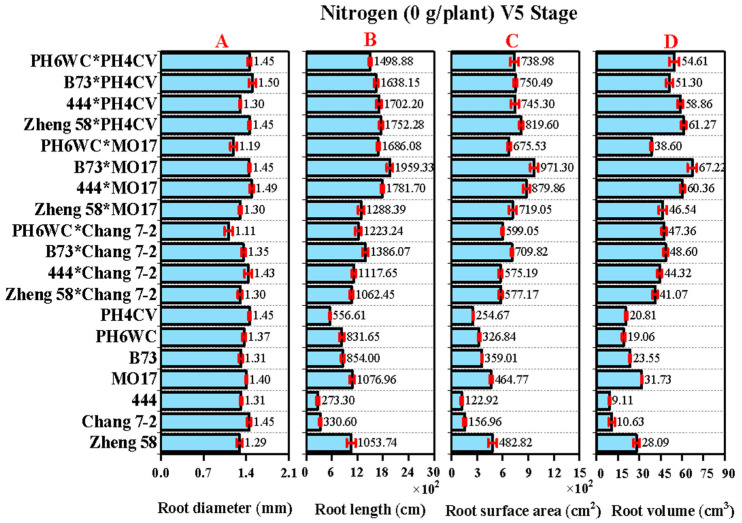
Root characteristics. (**A**) Root diameter (mm), (**B**) root length (cm), (**C**) root surface area (cm^2^), and (**D**) root volume (cm^3^) of maize genotypes at V5 stage under no N supply.

At the V5 stage, root traits such as root diameter (RD), root length (RL), root surface area (RSA), and root volume (RV) were assessed under no nitrogen supply, revealing the genetic basis of root architecture in maize genotypes. RD exhibited minimal variation, suggesting that early root expansion was predominantly genetically determined. By contrast, RL, RSA, and RV, which are key traits for nutrient and water absorption, showed significant variation among genotypes (Figure 1A–D). Hybrids B73 × MO17, 444 × MO17, and Zheng58 × PH4CV displayed higher RL and RSA. The larger RV observed in hybrids compared to inbred lines suggested enhanced NUpE due to increased access to soil resources.

### 2.3. Genotypic Variation in Primary Root Characteristics at V7 Stage

**Figure 2:**
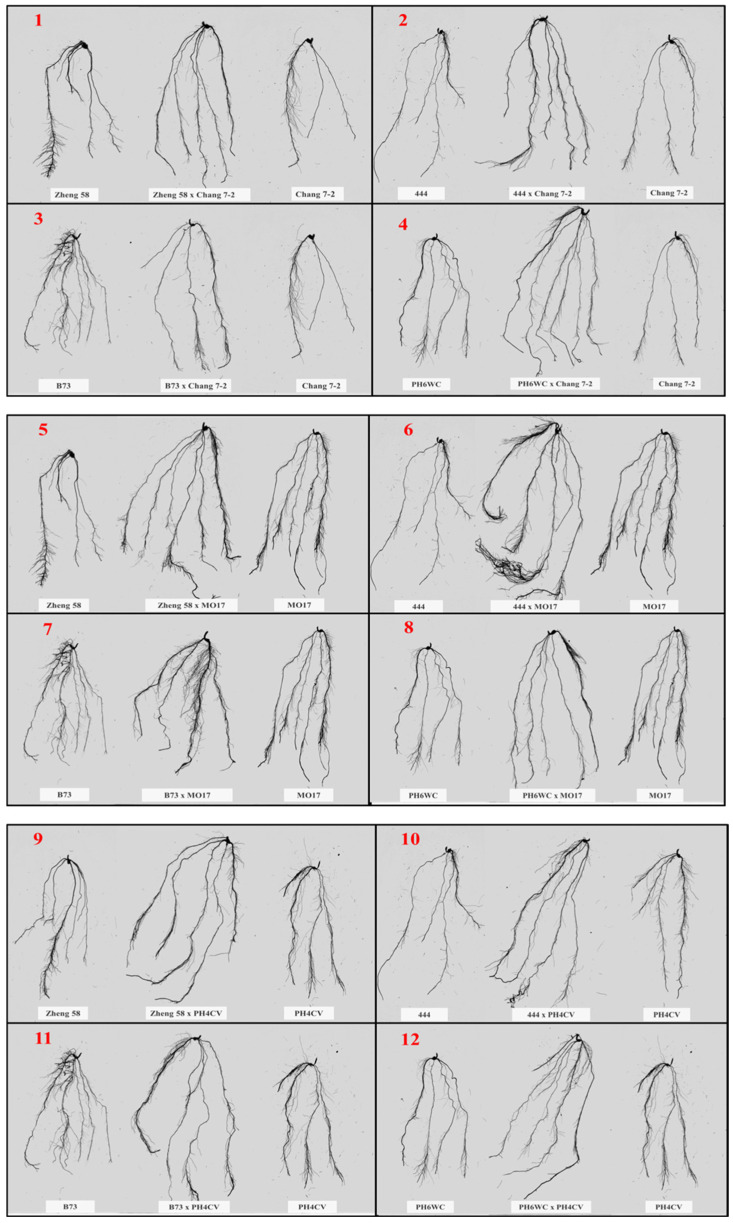
Comparison of primary root development in inbred lines and their hybrids (**1**–**12**) at the V7 growth stage. Each image displays the primary root of inbred lines (left and right) and their hybrid (center).

Nitrogen availability significantly influenced root traits at the V7 stage (Supplementary Figure S2), with hybrids exhibiting stronger responses than inbred lines (Figure 2). Nitrogen supplementation, particularly at the higher rate of 0.46 g N/plant, enhanced root growth by increasing root length (RL), root surface area (RSA), and root volume (RV). Among hybrids, Zheng 58 × PH4CV, B73 × MO17, and 444 × MO17 exhibited higher RL (652.30, 659.43, and 648.38 cm, respectively), RSA (341.66, 314.43, and 324.59 cm^2^, respectively), RV (69.53, 66.17, and 64.32 cm^3^, respectively), and RD (1.19, 1.13, and 1.33 mm, respectively). Among inbred lines, MO17 and B73 showed the highest RL (577.25 and 573.47 cm), RSA (308.29 and 291.73 cm^2^), RV (56.98 and 48.95 cm^3^), and RD (1.09 and 1.08 mm), respectively.

### 2.4. N-Assimilating Enzymes of N Metabolism and Metabolites

**Figure 3:**
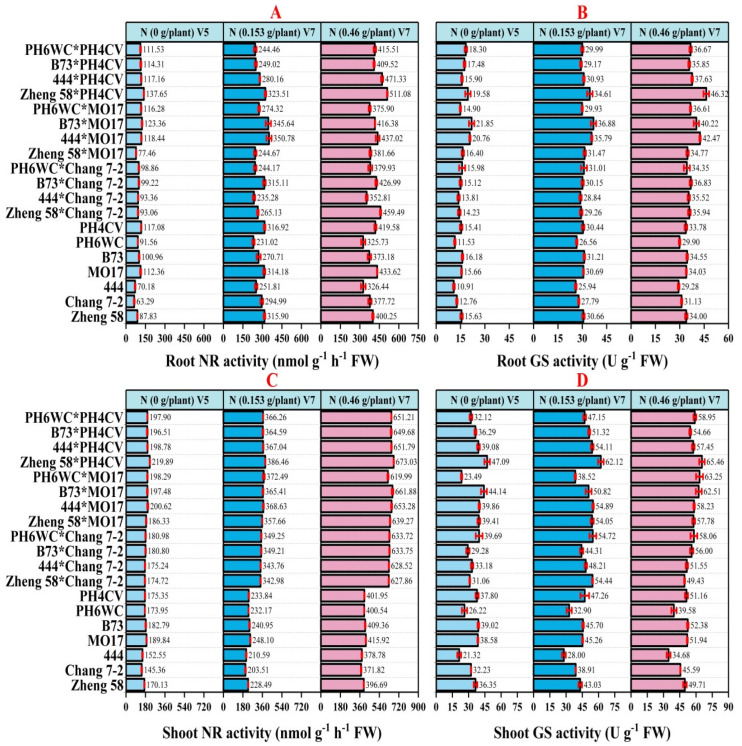
Impact of N on N-assimilating enzymes of maize genotypes. (**A**) Root NR activity (nmol g^−1^ h^−1^ FW), (**B**) root GS activity (U g^−1^ FW), (**C**) shoot NR activity (nmol g^−1^ h^−1^ FW), and (**D**) shoot GS activity (U g^−1^ FW), at V5 and V7 growth stages.

After uptake, N assimilation is facilitated by a range of enzymes. Genotype, nitrogen availability, and genotype × nitrogen (G × N) interaction had highly significant effects on root and shoot NR and GS enzyme activities (Figure 3). At the V5 stage, enzymatic activities were relatively low due to the absence of N, but at the V7 stage, both root and shoot NR and GS activities increased significantly (Figure 3A–D), particularly at 0.46 g N/plant. Among hybrids, Zheng 58 × PH4CV, B73 × MO17, and 444 × MO17 exhibited the highest root and shoot NR and GS enzyme activities, demonstrating superior NR and GS activity in both roots and shoots, which suggested an enhanced capacity for N assimilation. Similarly, among inbred lines, the highest root and shoot NR and GS activities were recorded in MO17, B73, PH4CV, and PH6WC. Additionally, metabolic markers such as nitrate content (NC), free amino acids (FAA), soluble protein (SP), and soluble sugars (SS) increased with higher nitrogen levels (Supplementary Figure S3), particularly in hybrids. The highest NC, FAA, and SP were recorded in hybrid Zheng 58 × PH4CV, followed by B73 × MO17 and 444 × MO17, while the highest SS was recorded in inbred line MO17.

### 2.5. Genotypic Variation in Physiological Traits

Leaf physiological traits, including chlorophyll content, chlorophyll fluorescence, photosynthetic rate (Pn), stomatal conductance (Gs), transpiration rate (Tr), and intercellular CO_2_ concentration (Ci), were significantly altered across different maize genotypes under varying N supply conditions (Supplementary Figures S4 and S5). The results indicated that chlorophyll content was significantly increased with N supply, particularly at higher N levels (0.46 g N/plant). Among hybrids, the highest chlorophyll contents were recorded in 444 × MO17 (2.18 mg/g FW), Zheng58 × MO17 (2.12 mg/g FW), and Zheng58 × PH4CV (2.11 mg/g FW). Among inbred lines, MO17 (1.97 mg/g FW), Zheng58 (1.86 mg/g FW), and B73 (1.78 mg/g FW) exhibited the highest values, reflecting superior nitrogen utilization. Hybrids Zheng58 × PH4CV, Zheng58 × MO17, and B73 × Chang 7-2 exhibited the highest carotenoid contents under the high N condition (Supplementary Figure S4).

Similarly, genotype, nitrogen, and genotype × nitrogen (G × N) interaction had highly significant effects on chlorophyll fluorescence (CF), Pn, Tr, Gs, and Ci under varying N supply conditions (Supplementary Figure S5). The results showed that Fv/Fm and Y (II) were significantly increased with N supply, particularly at higher N levels (0.46 g N/plant). Among hybrids, the highest Fv/Fm and Y (II) were recorded in Zheng58 × PH4CV (0.72 and 0.62, respectively), 444 × MO17 (0.68 and 0.60, respectively), and B73 × MO17 (0.64 and 0.58, respectively). Among inbred lines, PH4CV (0.59 and 0.58), MO17 (0.58 and 0.52), and B73 (0.52 and 0.51) showed the highest values for Fv/Fm and Y (II), respectively, under higher N supply. For Pn, E, and Gs, the highest values were recorded in hybrids Zheng58 × PH4CV (26.39, 7.34, and 305.69, respectively), PH6WC × Chang 7-2 (24.13, 7.69, and 318.57, respectively), and B73 × MO17 (23.67, 7.12, and 289.65, respectively). Among inbred lines, the highest values for Pn, Tr, and Gs were observed in MO17 (21.95, 7.04, and 296.71, respectively), PH4CV (22.86, 6.84, and 286.57, respectively), and B73 (21.82, 6.81, and 268.87, respectively) (Supplementary Figure S5). Finally, Ci decreased with increasing N levels, particularly at the V7 stage, indicating more efficient CO_2_ fixation under N-rich conditions.

### 2.6. Genotypic Variation in Morphological Traits

The maize genotypes exhibited significant variation in growth parameters in response to different N levels at the V5 and V7 stages (Supplementary Figure S6). Leaf area (LA) and plant height (PH) increased notably with N supply, particularly at the V7 stage, where the highest N levels led to substantial improvements in both parameters. Genotypes such as Zheng 58 × PH4CV (908.38 cm^2^ and 93.17 cm) and 444 × MO17 (848.46 cm^2^ and 94.47 cm) exhibited superior growth than other hybrids. Root dry weight (RDW), shoot dry weight (SDW), and total dry weight (TDW) were positively correlated with N availability, with the highest biomass accumulation observed at V7 under 0.46 g N/plant. Among hybrids, the highest RDW, SDW, and TDW were recorded in Zheng 58 × PH4CV (5.47 g, 16.22 g, and 21.69 g, respectively), followed by 444 × MO17 (6.37 g, 13.82 g, and 20.19 g, respectively). Among inbred lines, the highest RDW, SDW, and TDW were recorded in PH4CV (2.94 g, 7.09 g, and 10.03 g, respectively), followed by MO17 (3.07 g, 6.73 g, and 9.81 g, respectively).

### 2.7. Plant Biomass N Concentration

Nitrogen had a significant impact on root, shoot, and total plant N concentrations under varying supply conditions (Supplementary Figure S7). Genotypic variation in N concentration across tissues was observed, with increasing N levels leading to greater increases in N concentration in inbred lines compared to hybrids. Differences in N concentration across maize genotypes were evident under both N treatments. For root N concentration (RNC), higher N supply consistently increased N concentration, with the highest responses observed in inbred lines such as Zheng58 (2.37%), Chang 7-2 (2.26%), and PH4CV (2.26%), compared to their respective hybrids. Similarly, shoot N concentration (SNC) showed marked increases with higher N levels, particularly in inbred lines like MO17 (3.65%), PH4CV (3.63%), and 444 (3.53%), suggesting these inbred lines had superior NUtE. Total N concentration (TNC) exhibited similar trends, with inbred lines such as PH4CV (5.90%), Zheng58 (5.79%), 444 (5.65%), and MO17 (5.61%) showing higher TNC under elevated N conditions, indicating their enhanced capacity to assimilate and distribute N efficiently under high N supply.

### 2.8. Genetic Variation in Kinetic Parameters Among Maize Genotypes

Shoot dry weight was considered a key trait for identifying N-efficient genotypes and was plotted as alpha (α) in response to various N levels, indicated by beta (β) (Table 1). Alpha (α) represents the maximum SDW, reflecting the genotype’s growth potential at optimal N levels. Genotypes with higher α values have a greater capacity to accumulate biomass under higher N availability. Beta (β) indicates how quickly SDW increases with increasing N supply, representing the sensitivity or responsiveness of the genotypes to N. Evaluation of the kinetic parameters for 19 maize genotypes, including 7 inbred lines and 12 hybrids, under varying N supply at the V7 stage, provided crucial insights into their NUE. Among inbred lines, B73 exhibited the highest α value (12.85 ± 2.56), reflecting its superior growth potential under N fertilization. However, MO17 demonstrated the best fit with an R^2^ value of 0.94, suggesting a strong correlation between N supply and SDW. The low β value for MO17 (0.096 ± 0.019) indicated that this inbred line reached its maximum growth potential at lower N inputs, highlighting its N-efficient characteristics.

Among hybrids, Zheng58 × PH4CV stood out with the highest α value of 22.22 ± 0.274, emphasizing its exceptional ability to accumulate biomass under high N conditions. The R^2^ value of 0.95 confirmed the robust relationship between N supply and SDW. Additionally, 444 × MO17 exhibited a relatively high α value of 17.16 ± 2.092, with an R^2^ of 0.81, suggesting it was another strong contender for NUE. Among hybrids, PH6WC × Chang 7-2 and Zheng58 × MO17 showed lower β values (0.027 ± 0.01 and 0.055 ± 0.01, respectively), indicating that these genotypes reached their maximum SDW at lower N levels, thus demonstrating N efficiency. By contrast, hybrids like B73 × PH4CV and PH6WC × PH4CV showed higher β values, suggesting that their SDW was more responsive to higher N supply.

### 2.9. Plant Component N Accumulation

Root, shoot, and total N accumulation per plant were evaluated across 19 maize genotypes, including 7 inbred lines and 12 hybrids, under two N supply levels (0.153 and 0.46 g/plant) at the V7 growth stage (Table 2). The results revealed significant variations among genotypes for root N accumulation (RNA), shoot N accumulation (SNA), and total N accumulation (TNA) across both N treatments. The hybrid Zheng 58 × PH4CV consistently ranked highest in TNA, with an average of 0.296 g/plant under low N and 0.489 g/plant under high N, followed by 444 × PH4CV and 444 × MO17, demonstrating superior NUtE in both root and shoot components. MO17 exhibited the best performance among inbred lines, while Chang 7-2 consistently ranked lowest in N accumulation. The clear differentiation in N accumulation among genotypes suggested that the hybrid Zheng 58 × PH4CV was the best-performing genotype in terms of N accumulation under varying N supply.

### 2.10. N Use Efficiency Indices

The nitrogen use efficiency indices, including N uptake efficiency (NUpE) and nitrogen utilization efficiency (NUtE), were recorded to assess the potential of the maize genotypes for efficient N utilization. Significant variations in NUE were observed among the maize genotypes (Table 3). NUE was influenced by both NUpE and NUtE, with notable differences between inbred lines and hybrids. In general, hybrids outperformed inbred lines in NUE. Under low N supply, hybrids such as Zheng 58 × PH4CV (94.16), 444 × PH4CV (93.14), and B73 × MO17 (93.84) exhibited the highest NUE values. Under high N supply, Zheng 58 × PH4CV (47.16) maintained its superior NUE, followed by 444 × PH4CV (40.38). Ranking the genotypes based on average NUE across both N levels revealed that Zheng 58 × PH4CV, 444 × PH4CV, and B73 × MO17 consistently exhibited higher NUE. These genotypes combined high NUpE and NUtE, making them the most effective across both N-limited and N-sufficient conditions. Notably, Zheng 58 × PH4CV exhibited the highest overall NUE, driven by its superior NUpE and NUtE, making it the most N-efficient hybrid.

### 2.11. Heterosis Among Maize Genotypes

The absolute heterosis (AH) and mid-parent heterosis (MPH) values demonstrated considerable enhancement in multiple parameters (Table 4). Root activity exhibited strong heterosis with 42.74% AH and 58.94% MPH, while RSA showed moderate improvement (15.39% AH, 34.10% MPH). Root and shoot NR activities displayed contrasting patterns: root NR showed negative AH (−2.56%) but positive MPH (4.44%), while shoot NR exhibited high heterosis (56.45% AH, 60.91% MPH). Both root and shoot GS activities showed positive heterosis, with root GS showing 8.40% AH and 12.57% MPH, and shoot GS showing 15.31% AH and 26.18% MPH. Chlorophyll a+b content demonstrated moderate heterosis (3.56% AH, 11.35% MPH), and Pn showed slight positive heterosis (0.16% AH, 3.56% MPH). Chlorophyll fluorescence parameters such as Fv/Fm and Y (II) also improved, with Fv/Fm showing 8.97% AH and 11.52% MPH, and Y (II) showing 6.60% AH and 10.91% MPH. Total dry matter exhibited strong heterosis (92.01% AH, 116.05% MPH), while TNC showed negative heterosis (−28.00% AH, −25.89% MPH). By contrast, total N accumulation displayed positive heterosis (35.99% AH, 56.29% MPH). The nitrogen use efficiency indices, including NUpE, NUtE, and NUE, also showed substantial heterosis, with NUE exhibiting the highest values (94.30% AH, 119.81% MPH), indicating superior N efficiency in hybrids.

### 2.12. Global ANOVA and Principal Component Analysis

#### 2.12.1. Global ANOVA

**Figure 4:**
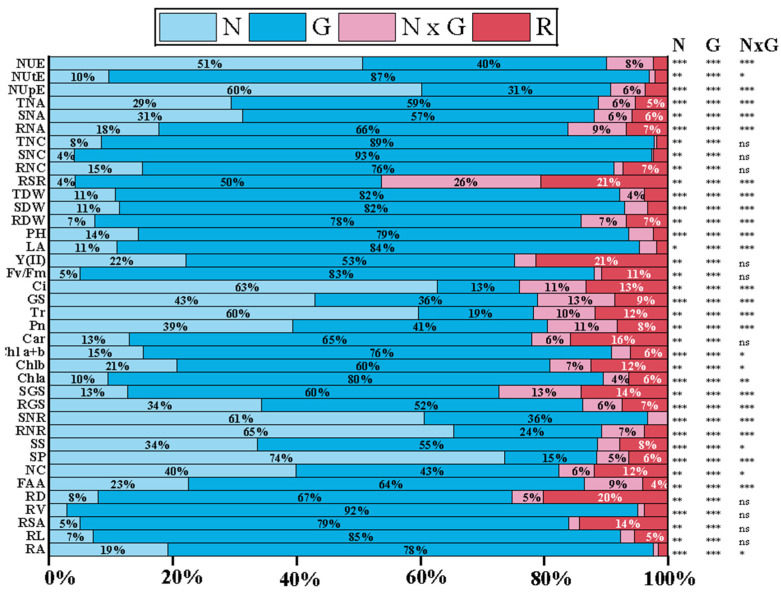
Global ANOVA of morphological, physiological, and biochemical traits of maize genotypes in response to N (0.153 and 0.46 g/plant) supply. The percentages of type III sums of squares indicate the contributions of N, genotypes (G), their interaction (N × G), and residuals (R). The *p*-values from the F-test are shown in the following manner: * *p*-value < 0.05; ** *p*-value < 0.01; *** *p*-value < 0.001; ns: not statistically significant. RA, root activity; RL, root length; RSA, root surface area; RV, root volume; RD, root diameter; FAA, free amino acid; NC, nitrate content; SP, soluble protein; SS, soluble sugar; RNR, root NR activity; SNR, shoot NR activity; RGS, root GS activity; SGS, shoot GS activity; Chl a, chlorophyll a; Chl b, chlorophyll b; chl a+b, chlorophyll a+b; Car, carotenoid content; Pn, photosynthetic rate; Tr, transpiration rate; GS, stomatal conductance; Ci, intercellular CO_2_ concentration; Fv/Fm, maximum photochemical efficiency; Y (II), photochemical quantum yield; LA, leaf area; PH, plant height; RDW, root dry weight; SDW, shoot dry weight; TDW, total dry weight; RSR, root-to-shoot ratio; RNC, root N concentration; SNC, shoot N concentration; TNC, total N concentration; RNA, root N accumulation; SNA, shoot N accumulation; TNA, total N accumulation; NUpE, N uptake efficiency; NutE, N utilization efficiency; NUE, N use efficiency.

The global analysis of variance revealed significant differences among genotypes and N supply for all traits (Figure 4). Nitrogen supply significantly influenced the studied indicators, accounting for 5% to 74% of the total observed variance. For certain traits, such as root NR activity, shoot NR activity, SP, Tr, Ci, NUpE, and NUE, N supply accounted for more than 50% of the overall variation. The genotypic effect was also statistically significant for all parameters, contributing to 13% to 93% of the total variation. The most substantial genetic variation was observed for RA, RL, RSA, RV, RD, chlorophyll a+b content, chlorophyll fluorescence parameters (Fv/Fm, Y (II)), TDW, TNC, TNA, and NUtE (Figure 4). Interaction effects between N and genotype were significant and played a crucial role in influencing these traits, accounting for 2% to 26% of the total variance. Notably, the highest interaction effect was observed for RSR, which explained 19% of the overall variance.

#### 2.12.2. Principal Component Analysis (PCA)

**Figure 5:**
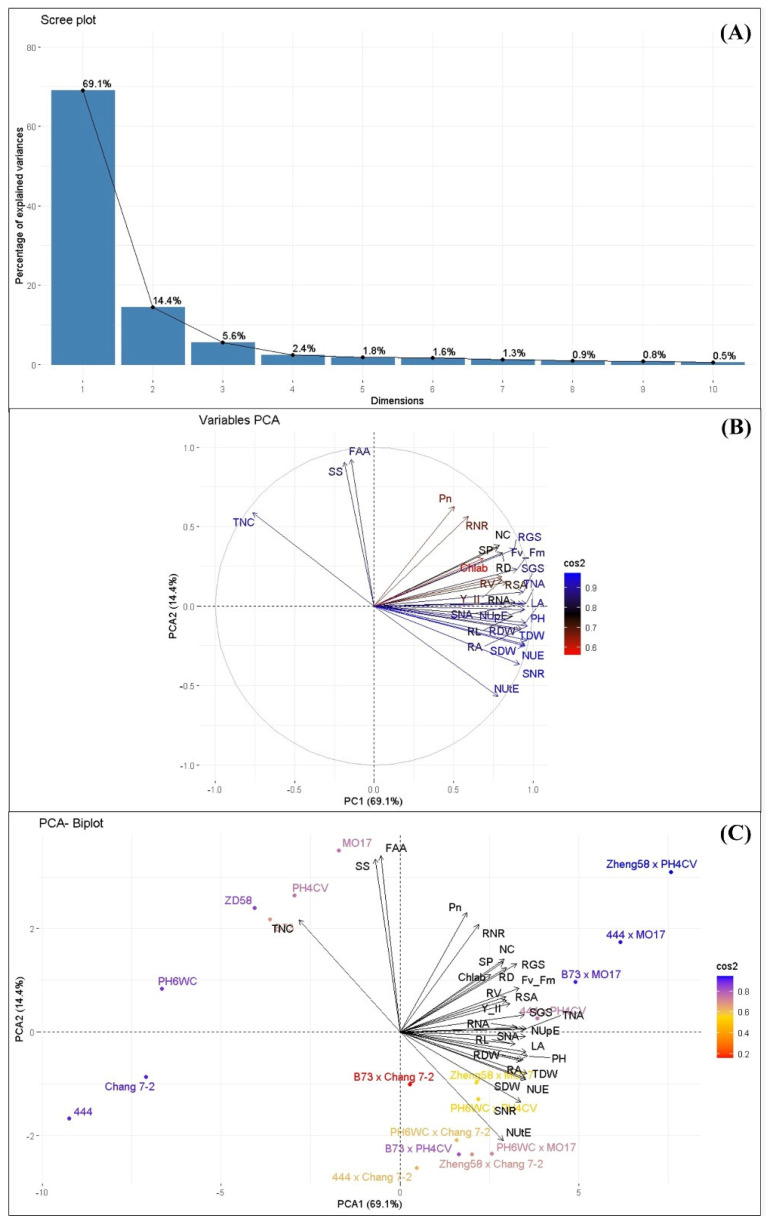
Principal component analysis. (**A**) Scree plot showing important principal components (PCs) for different traits in 19 genotypes; (**B**) variable PCA plot showing the relationships among 32 different traits in 19 genotypes; (**C**) PCA biplot displaying different traits and 19 individuals inbred line on PCA1 and PCA2.

Principal component analysis was conducted to identify the key principal components associated with desirable traits in maize genotypes (Figure 5A–C). PCA allowed for the assessment of correlations between different traits and the identification of key components explaining the observed variation. The first three principal components (PC1, PC2, and PC3) were selected for interpretation, as they collectively accounted for 89.13% of the total variance among the 19 maize genotypes. Specifically, PC1 explained 69.1%, PC2 accounted for 14.4%, and PC3 contributed to 5.6% of the total variance (Figure 5A). In the PCA variable plot, the cos^2^ values of each variable represented the proportion of variance explained by the principal components (Figure 5B). Variables with high cos^2^ values, indicated by blue color, exhibited strong correlations with the principal components and contributed significantly to the overall variance. By contrast, variables with low cos^2^ values, shown in red, had minimal influence on the variance. Similarly, genotypes appearing in blue on the PCA plot tended to have high cos^2^ values, indicating strong correlations with PC1 and significant contributions to the total variance. Among hybrids, Zheng58 × PH4CV had the highest cos^2^ value, followed by 444 × MO17 and B73 × MO17. PH6WC showed the highest cos^2^ value, followed by PH4CV and MO17, marking them as the best-performing inbred lines. PC1 explained 69.1% of the total variance, with key traits contributing to this component (Figure 5C). High-loading variables for PC1 included TDW (0.215), PH (0.215), TNA (0.214), NUE (0.213), LA (0.213), NUpE (0.211), SGS activity (0.211), SNR activity (0.210), and RA (0.210). PC2, which explained 14.4% of the variation, was influenced by traits such as FAA (0.45), Pn (0.30), TNC (0.28), NutE (−0.27), and RNR (0.27).

### 2.13. Elucidating Pathways from Root Traits to NUE: A Comprehensive Structural Equation Modeling (SEM) Approach in Maize Genotypes

**Figure 6:**
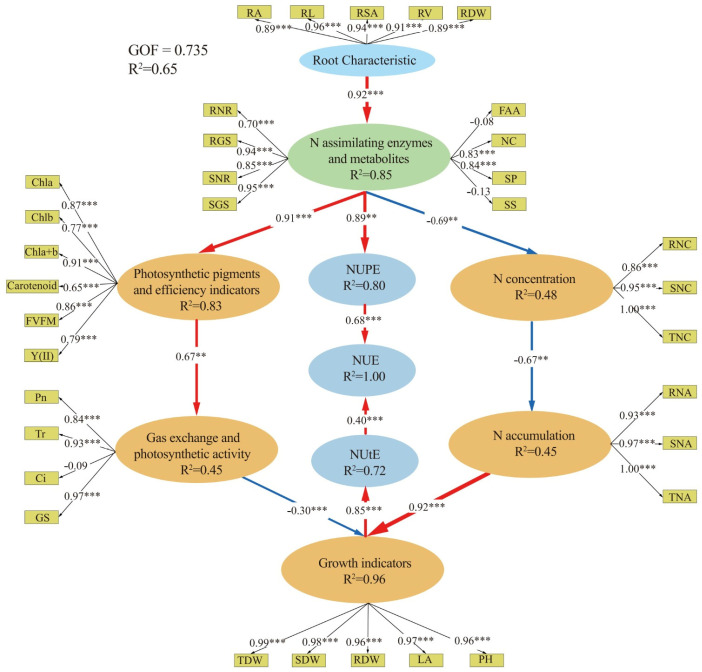
Structural equation model (SEM) explaining the direct and indirect effects of root characteristics, N−assimilating enzymes and metabolites, photosynthetic pigments and efficiency indicators, tissue N concentration, gas exchange and photosynthetic activity, N accumulation, growth indicators, NUpE, and NutE on NUE. Red lines represent significant positive relationships, while blue indicates non-significant relationships. The thickness of the line indicates the strength of the causal relationship, accompanied by the standardized path coefficient. The outer model shows the loading values of indicators on latent variables, with solid and dashed lines representing significant and non-significant relationships, respectively. R^2^ represents the variance explained by the dependent variables in the SEM, and GOF indicates the goodness of fit of the entire model. The significance levels for each predictor variable are ** *p* < 0.01; and *** *p* < 0.001.

The simulation results showed that the combined reliability value and Cronbach α coefficient of the model surpassed the recommended threshold of 0.70. The average variance extracted value exceeded 0.50 (Figure 6). The SEM results demonstrated significant hierarchical relationships among root characteristics (RA, RL, RSA, RV, and RD), N-assimilating enzymes and metabolites (NR, GS, FAA, NC, SP, and SS), NUpE, photosynthetic pigments and efficiency (chl a, b, a+b, carotenoid, Y (II), Fv/Fm), gas exchange and photosynthetic activity (Pn, E, Gs, Ci), N concentration (root, shoot, and total N concentration), N accumulation (RNA, SNA, TNA), growth indicators (LA, PH, RDW, SDW, and TDM), NutE, and NUE.

The SEM explained 85% of the variance in N-assimilating enzymes and metabolites by root characteristics; 83% of photosynthetic pigments, 48% of tissue N concentration, and 80% of NUpE by N metabolism enzymes and metabolites; 45% of gas exchange and photosynthetic activity by photosynthetic pigments; 45% of N accumulation by leaf N concentration; 96% of plant growth by gas exchange, photosynthetic activity, and N accumulation; 72% of NUtE by growth indicators; and 100% of NUE is by NutE and NUpE. NUE was directly increased with NUpE and NUtE. The path coefficients were 92% (N-assimilating enzymes and metabolites), 91% (photosynthetic pigments and efficiency), 89% (NUpE), 67% (gas exchange and photosynthetic activity), −69% (N concentration), −67% (N accumulation), −30 and 92% (growth indicators), 85% (NUtE), and 40% and 68% (NUE).

### 2.14. Regression and Correlation Analysis of TDM/NUpE/NutE and Agronomic, Root, and NUE-Related, and Physiological Traits

#### 2.14.1. Regression and Correlation of TDM and Root, Agronomic, and Physiological Traits

The relationships between total dry matter (TDM) and various root, agronomic, and physiological traits in maize genotypes under different N conditions are summarized in Supplementary Table S1. Under high N supply, TDM exhibited strong positive correlations with RA (r = 0.86 ***), RL (r = 0.77 ***), RSA (r = 0.68 ***), RDW (r = 0.95 ***), RNR activity (r = 0.63 ***), and RGS activity (r = 0.82 ***), suggesting that these traits were crucial for biomass accumulation. Similarly, under low N conditions, TDM showed significant correlations with RA (r = 0.84 ***), RL (r = 0.70 ***), RSA (r = 0.54 ***), and RGS activity (r = 0.51 ***), further emphasizing the importance of root traits in sustaining plant growth under nutrient-limiting conditions. Notably, SDW exhibited near-perfect correlations with TDM under both high (r = 0.99 ***) and low N (r = 0.98 ***), demonstrating its role as a reliable predictor of total plant biomass. These findings highlighted that root and shoot development traits were key indicators of TDM under varying N availability. Additionally, TDM showed strong positive correlations with RNA (r = 0.90 ***), SNA (r = 0.91 ***), and TNA (r = 0.95 ***). Interestingly, negative correlations were observed between TDM and root SP (r = −0.27 *) and FAA (r = −0.33 *) under high N conditions, with a more pronounced negative relationship between TDM and root SS under low N (r = −0.38 **).

#### 2.14.2. Regression and Correlation of NUpE and Root, Agronomic, and Physiological Indicators

Under high N conditions, NUpE showed significant positive correlations with RA (r = 0.80 ***), RL (r = 0.73 ***), RSA (r = 0.67 ***), RNR activity (r = 0.70 ***), RGS activity (r = 0.84 ***), and RDW (r = 0.92 ***), highlighting the critical role of efficient root systems in enhancing N uptake (Supplementary Table S2). Similarly, under low N supply, NUpE exhibited strong positive correlations with RA (r = 0.78 ***), RL (r = 0.62 ***), RGS activity (r = 0.53 ***), and RDW (r = 0.91 ***), underscoring the maize plant’s ability to maintain NUpE even under nutrient-limiting conditions. Notably, the correlation between NUpE and RD was stronger under low N (r = 0.58 ***) than under high N (r = 0.52 ***), suggesting that the ability of maize genotypes to maintain greater RD in nutrient-poor environments was essential for effective N uptake. These results highlighted the importance of root architecture, particularly RA and RSA, in enhancing NUpE, especially under limited N availability. Negative correlations were observed for root FAA (r = −0.13) and root SS (r = −0.09) under high N, with a stronger negative association for root SS under low N (r = −0.21).

#### 2.14.3. Regression and Correlation of NUtE and Root, Agronomic, and Physiological Traits

Strong positive correlations were observed between NUtE and RA (r = 0.78 ***), RL (r = 0.67 ***), RDW (r = 0.75 ***), SNR activity (r = 0.90 ***), SGS activity (r = 0.56 ***), LA (r = 0.73 ***), SDW (r = 0.82 ***), RNA (r = 0.64 ***), and SNA (r = 0.55 ***) under high N conditions. Under low N conditions, NUtE remained strongly correlated with RA (r = 0.78 ***), RL (r = 0.68 ***), RDW (r = 0.67 ***), SNR activity (r = 0.86 ***), SGS activity (r = 0.59 ***), SDW (r = 0.86 ***), and SNA (r = 0.64 ***), suggesting that above-ground biomass production was a reliable indicator of NUtE (Supplementary Table S3). Additionally, root-free amino acids (r = −0.63 ***) and root SS (r = −0.56 ***) showed significant negative correlations with NUtE under high N. These negative correlations persisted under low N for root FAA (r = −0.47) and root SS (r = −0.61 ***), indicating that a reduction in these components could enhance NUtE.

## 3. Discussion

Many plants have adaptive responses to different environments, including changes in behavior due to N availability [49,50]. The unavailability of N often results in reduced growth [51], N metabolism [37], photosynthetic activity [52], biomass [53], N translocation [53,54], N accumulation [54,55], and NUE [36].

Root activity and morphology, including RL, RSA, RV, and RD, are critical in determining a genotype’s NUpE and overall NUE. In this study, hybrids showed significantly higher root activity and enhanced root morphology under high N conditions (Supplementary Figures S1 and S2), which aligned with the findings in [51,56], where increased root morphology improved nutrient uptake, directly enhancing NUE. NUpE showed highly significant positive correlations with RA (r = 0.80 *), RL (r = 0.73 ***), RSA (r = 0.67 ***), RV (r = 0.66 ***), RD (r = 0.52 ***), and RDW (r = 0.92 ***) (Supplementary Table S2). Effective root architecture, particularly in hybrids, enhanced nutrient uptake under both high and low N conditions [57], as evidenced by hybrids such as Zheng58 × PH4CV, 444 × MO17, and B73 × MO17, which exhibited optimal root morphology compared to inbred lines. The importance of RL and RSA for nutrient scavenging was reinforced in [58], where genotypes with greater RL and RSA are better adapted to low-N environments, as these traits enabled a broader range of nutrient absorption. Additionally, heterotic effects in root traits among hybrids contributed significantly to improved NUpE, particularly in environments with variable N availability [57], aligning with our findings on the superior root system functionality of hybrids under both high and low N inputs.

Higher values for morphological traits such as PH, LA, RDW, and SDW were observed in hybrids Zheng58 × PH4CV, 444 × MO17, and B73 × MO17 compared to inbred lines B73, MO17, Zheng58, and 444 (Supplementary Figure S6). These increases in morphological traits in hybrids were attributed to improved N uptake (Table 3), greater N metabolism enzyme activity (Figure 3), and superior photosynthetic activity (Supplementary Figure S5). Total dry matter showed highly significant positive correlations with NUpE (r = 0.95 *) and NUtE (r = 0.81 ***). These findings aligned with studies on maize [39], rice [43], and wheat [59], where efficient genotypes demonstrated superior performance. Genotypic effects, as determined by global ANOVA (Figure 4), accounted for 13–93% of the total variance, indicating significant variation among maize genotypes across all traits.

Increasing the NUE is essential for maintaining high productivity levels with comparatively low N supply [60]. Identifying the genotypic responses to varying N conditions is key to understanding the mechanisms and traits contributing to NUE. NR and GS are critical enzymes in N assimilation, as they facilitate the conversion of absorbed N into amino acids and proteins. Our results showed significant increases in root and shoot NR and GS activities in hybrids such as Zheng58 × PH4CV, 444 × MO17, and B73 × MO17 at V7 under different N treatments (Figure 3). The contrasting patterns of N uptake, utilization, and accumulation among the genotypes were further supported by differences in their N-assimilating enzymes and growth responses at the V7 stage (Table 3 and Table 4). NUpE and NUtE were highly positively correlated with root NR (r = 0.70 *, r = 0.35 ***), shoot NR (r = 0.78 ***, r = 0.90 ***), root GS (r = 0.84 ***, r = 0.53 ***), and shoot GS (r = 0.69 ***, r = 0.56 ***), respectively. These findings were in agreement with those reported in [37,55,61], where N-efficient hybrids possessed higher NR and GS activities than N-inefficient ones under N-sufficient conditions. Higher NR and GS activities also benefit N accumulation (Table 2) and distribution in plants, as they improve the N assimilation and metabolism rate, thereby enhancing NUE [36]. Nitrogen-assimilating enzyme activities in different genotypes directly correlated with N availability and assimilation efficiency [62]. High NR and GS activities observed in hybrids like Zheng58 × PH4CV, 444 × MO17, and B73 × MO17 highlighted their increased capacity for N assimilation, reflecting the physiological advantages of improved N assimilation pathways in maize hybrids.

Moreover, strong positive correlations were observed between NUpE and root NC (r = 0.70 *) and root SP (r = 0.52 ***) under high N supply. Genotypic differences in N metabolism were further evidenced by variations in root NC and root SP, with hybrids such as Zheng58 × PH4CV, 444 × MO17, and B73 × MO17 exhibiting higher root NC and root SP compared to inbred lines (Supplementary Figure S3). Substantial decreases in both root NC and root SP were observed under low N supply, which may have been due to impaired N uptake disrupting amino acid and protein synthesis [63]. This reduction in N assimilation hampers enzyme production, which is essential for these processes, and decreases chlorophyll levels, thus reducing photosynthesis and the energy available for protein synthesis [64]. N deficiency also triggers stress responses that degrade existing proteins and limit overall plant growth, further diminishing protein and amino acid accumulation in leaves [65]. These findings highlight the importance of NR, GS, and metabolites as key parameters in N metabolism [37,66].

Photosynthesis is highly responsive to N availability [67], playing a crucial role in the synthesis of photosynthetic apparatus and enzymes [68,69]. N significantly affected chlorophyll indices, chlorophyll fluorescence, and gas exchange indicators (Supplementary Figures S4 and S5), consistent with previous maize studies [52,70]. Similarly, low N levels significantly reduced photosynthetic activity in maize genotypes, in line with findings in maize [52,71], wheat [72], and rice [73]. The decrease in photosynthetic activity under low N conditions can be attributed to impaired carboxylation, as indicated by elevated intercellular CO_2_ concentrations [67]. Previous studies have shown strong positive correlations between NUpE and NutE with various photosynthetic indices [28]. Our study similarly demonstrated significant correlations between NUpE and NutE with photosynthesis indices. NUpE was positively correlated with chl a+b (r = 0.56 *), Pn (r = 0.46 ***), Fv/Fm (r = 0.82 ***), and Y (II) (r = 0.68 ***) (Supplementary Table S2). NutE also exhibited positive correlations with chl a+b (r = 0.43 ***), Pn (r = 0.10), Fv/Fm (r = 0.52 ***), and Y (II) (r = 0.40 ***) (Supplementary Table S3), consistent with findings on N-efficient genotypes [23,44]. Conversely, reduced photosynthetic activity was likely a result of photosystem inhibition, as indicated by the downregulation of genes under contrasting N conditions [74,75].

Previous studies on maize have established strong relationships among N management, N metabolism, accumulation, and translocation [25,37,51,54,55]. N accumulation across root, shoot, and total plant tissues was notably higher in hybrids, suggesting enhanced N assimilation capabilities than inbred lines (Table 2). Our findings aligned with previous research that observed that NUE heterosis in maize hybrids often resulted from an improved capacity for N uptake and partitioning within the plant [27]. The higher N accumulation in hybrids, particularly Zheng58 × PH4CV, 444 × PH4CV, and 444 × MO17 under high N, supports the notion that hybrids generally exhibit higher N accumulation than inbred lines, which is crucial for sustaining growth under both N-sufficient and N-deficient conditions [19,35,57]. N accumulation, such as RNA and SNA, was strongly positively correlated with NUpE (r = 0.90 ***, r = 0.98 ***) and NutE (r = 0.64 ***, r = 0.55 ***), respectively (Supplementary Table S2). These results aligned with previous findings that maize hybrids with superior NUE showed increased N accumulation due to efficient N uptake and utilization [27,76].

This research provides comprehensive insights into NUE indices, including NUpE, NutE, and overall NUE, across genotypes (Table 3, Supplementary Tables S2 and S3). The high NUE exhibited by hybrids such as Zheng58 × PH4CV, 444 × PH4CV, and B73 × MO17 aligned with previous studies showing that hybrids maintained higher NUE by optimizing both N uptake and utilization, which is particularly advantageous under low N conditions [77]. Additionally, the heterosis for NUE observed in these maize hybrids can be attributed to efficient N uptake and utilization, supporting our findings of superior NUE in hybrids [27]. The efficient N metabolism in these hybrids, characterized by the continuous conversion of nitrate into N-containing compounds, highlights the well-coordinated system of N uptake, transport, and assimilation, which enhances NUE.

This study focuses on early vegetative growth stages, limiting insights into NUE across the full maize growth cycle, particularly during reproductive phases. Also, conducting the experiments in pots, while reducing variability, does not fully reflect field environments with diverse soil, temperature, and stress factors. The use of only two nitrogen treatment levels may not capture the full spectrum of plant responses. Additionally, soil microbial interactions and advanced computational tools, such as modeling and machine learning for predicting NUE, were not explored, highlighting the need for further validation and extension of these findings. Similarly, an interdisciplinary framework integrating genetics, physiology, and soil science will provide a comprehensive understanding of NUE, leading to more efficient maize hybrids and sustainable nitrogen management practices.

## 4. Materials and Methods

### 4.1. Plant Materials and Experimental Design

In the present study, 7 inbred lines and their 12 hybrids were used as test materials during the early vegetative growth stages (Table 5). The experiment was conducted from May to July 2024 in the greenhouse of Northeastern Agricultural University, Harbin, Heilongjiang, China. All inbred lines were crossed in 2023 to produce F1 hybrids. In May 2024, a two-way randomized complete block design (RCBD) pot experiment was carried out to evaluate the F1 direct crosses and their respective inbred lines as Factor A, with nitrogen (N) treatments at two levels (0.153 and 0.46 g/plant) as Factor B. No N was applied to any genotype during the V5 growth stage. However, N treatments were applied at the V7 growth stage, with the designated amounts of 0.153 g/plant and 0.46 g/plant.

### 4.2. Measurements of Plant Traits

#### 4.2.1. Root Characteristics

Root characteristics, including root activity (RA), root length (RL), root surface area (RSA), root volume (RV), root diameter (RD), root dry weight (RDW), shoot dry weight (SDW), total dry weight (TDW), and root-to-shoot ratio (RSR), as well as plant growth indicators such as plant height (PH) and leaf area (LA), were measured at the V5 and V7 growth stages.

Root activity was determined using the triphenyl tetrazolium chloride (TTC) method [78]. Root system images were obtained using a root scanner (Microtek ScanMaker i800 Plus, Shanghai Zhongjing Technology Co., Ltd., Shanghai, China). RL, RSA, RV, and RD were analyzed using a root analysis system (Hangzhou Wanshen Test Technology Co., Ltd., Hangzhou, China). Plant height was measured by selecting three plants from each treatment. Leaf length and maximum leaf width were measured with a measuring tape, and LA was calculated using the formula: leaf area = 0.75 × leaf length × maximum leaf width. Root and shoot dry weights were measured using an electronic analytical balance after drying the samples in an oven at 105 °C for 24–48 h (AEL-160-21, Shimadzu Corporation, Kyoto, Japan).

#### 4.2.2. Measurement of N-Assimilating Enzymatic Activities

Enzymatic activity at the V5 and V7 growth stages was measured using assay kits from Grace Biotechnology Company (Suzhou, China) and analyzed with a microplate reader (Thermo Scientific Multiskan FC, Waltham, MA, USA).

The nitrate reductase (NR) activity of roots and shoots was evaluated following the methodology described in [78], with results reported as nmol h^−1^g^−1^ fresh weight. Fresh shoot sample (0.1 g) was depigmented twice by adding 1 mL of 80% ethanol, followed by homogenization in an ice bath and centrifugation at 4 °C (12,000 rpm, 10 min), while root samples did not need a depigmentation process. The supernatant was discarded and 1 mL of extraction buffer was added to the tube. The sample was then homogenized and centrifuged under the same conditions to produce the crude enzyme extract. For the NR assay, reaction mixtures were prepared in a 96-well plate with samples, reagent 1, and reagent 2 (80, 280, and 120 μL, respectively). Control tubes contained sample and distilled water (80 and 400 μL, respectively). After a 30 min incubation at 30 °C in the dark, the reaction mix (400 μL) was added, thoroughly mixed, and the sample was incubated in the dark for 15 min before measuring the NR activity at 530 nm using a microplate reader.

The glutamine synthetase (GS) activity of roots and shoots was determined using the protocol described in [78]. Root or shoot sample (0.1 g) was treated with 1 mL of extraction buffer, homogenized in an ice bath, and then centrifuged at 4 °C (12,000 rpm, 10 min). The resulting supernatant, containing the crude enzyme extract, was stored on ice until further testing. In a 96-well plate, assay tubes received sample, reagent 1 (200 μL), reagent 2 (320 μL), and reagent 3 (120 μL), while control tubes contained sample, reagent 1 (200 μL), and reagent 3 (120 μL). After a 30 min ice bath at 37 °C, reagent 4 (200 μL) was added to both assay and control tubes. Following a 2 min reaction, samples were centrifuged at 4 °C (8000 rpm, 10 min), and the GS activity was measured at 540 nm using a microplate reader.

#### 4.2.3. Determination of Root Soluble Protein (SP), Free Amino Acids (FAA), Nitrate Content (NC), and Soluble Sugars (SS)

The Bradford method [79], which utilizes Coomassie Brilliant Blue (G-250) dye and albumin as a reference standard [80], was used to determine the root soluble protein content. Root sample (0.5 g) was ground in liquid N and homogenized with distilled water, followed by centrifugation at 10,000 rpm for 10 min at 4 °C. One mL of the supernatant was mixed with 5 mL of Coomassie Brilliant Blue (G-250) solution in a tube, and the absorbance was measured at 450 nm.

The free amino acid (FAA) content was determined using a modified ninhydrin method [81,82]. Fresh root sample (0.5 g) was ground with 5 mL of 10% acetic acid and then diluted to 100 mL with distilled water. The resulting extract was filtered and 1 mL of the filtrate was mixed with 1 mL of distilled water, 3 mL of ninhydrin solution, and 0.1 mL of ascorbic acid in a test tube. The mixture was heated in boiling water for 15 min, cooled rapidly, and shaken until a blue-purple color developed. Finally, 4.9 mL of absolute ethanol was added and thoroughly mixed. The absorbance was then measured at 580 nm using a UV-5500 spectrophotometer (Shanghai Chemical Laboratory Equipment Co., Ltd., Beijing, China).

The soluble sugar (SS) content was determined using the Anthrone method [83]. Fresh root sample (0.3 g) was homogenized with 5 mL of ethanol, followed by centrifugation for 10 min. To analyze the sugar content, 3 mL of anthrone reagent (150 mg anthrone dissolved in 100 mL concentrated H_2_SO_4_) was mixed with 0.1 mL of the supernatant. The mixture was incubated in a boiling water bath for 10 min and the absorbance was measured at 630 nm using a UV-5500 spectrophotometer. Concurrently, the nitrate content in fresh samples was determined using the salicylic acid method outlined in [84].

#### 4.2.4. Chlorophyll (Chl) and Carotenoid (Car) Contents

At the V5 and V7 growth stages, 0.1 g of leaf FW (fresh weight) was crushed in 95% ethanol. The obtained extract was transferred to centrifuge tubes and incubated for 48 h at a temperature of 4 °C in the absence of light. A UV-5500 spectrophotometer was used to measure the absorbance of the supernatant at wavelengths of 470, 649, and 665 nm. The chlorophyll content was measured using the equations provided in [85]. Each measurement was performed three times to ensure statistical reliability.

Chl a = (13.95 × D665 − 6.88 × D649)(1)

Chl b = (24.96 × D649 − 7.32 × D665)(2)

Car = (1000 − D470 − 2.05 × Ca − 114.8 × Cb)/245(3)

#### 4.2.5. Gas Exchange and Chlorophyll Fluorescence (CF)

The net photosynthetic rate (Pn), transpiration rate (Tr), stomatal conductance (Gs), and intercellular CO2 concentration (Ci) of expanded and clean leaves at the V5 and V7 stage were measured from 9:00 to 11:30 a.m. using a Yaxin-1101 photosynthesis tester. Furthermore, CF indicators such as maximum photochemical efficiency (Fv/Fm) and quantum yield of photosystem II [Y (II)] were measured on the middle part of the leaves using a chlorophyll fluorometer (PhotosynQ, MultispeQ fluorometer developed by PhenoTrait Technology Co., Ltd., Waltham, MA, USA).

#### 4.2.6. Measurement of N Concentration, N Accumulation, and N Efficiency Indices

At the V7 stage, the plant N content in various tissues (root and shoot) was analyzed using the Kjeldahl method, involving digestion, distillation, and titration. Samples were dried, ground, and digested with H_2_SO_4_-H_2_O_2_. The resulting digest was distilled and titrated to determine the root N concentration (RNC), shoot N concentration (SNC), and total N concentration (TNC). The N content in each fraction was calculated by multiplying the N concentration by the biomass of that fraction. Root N accumulation (RNA), shoot N accumulation (SNA), and total N accumulation (TNA) were determined by multiplying the N concentration of each plant tissue by its total dry weight. The performance of the genotypes was analyzed by directly comparing the values of α and β in a way that is similar to the well-known Michaelis–Menten enzyme kinetics Vmax and Km [86].

Nitrogen use efficiency (NUE) is defined as the amount of biomass produced per unit of nitrogen available to the plant, as described in [87]. NUE has two primary components: (1) the efficiency of nitrogen absorption (uptake), and (2) the efficiency with which the absorbed nitrogen is utilized to produce biomass. These are expressed as follows:

(4) $$NUpE=\frac{Nt}{Ns}$$

where *NUpE*, nitrogen uptake efficiency; *Nt*, total N accumulation at V7; *Ns*, nitrogen supply.

(5) $$NUtE=\frac{Bw}{Nt}$$

where *NutE*, nitrogen utilization efficiency; *Bw*, biomass weight at V7; *Nt*, total nitrogen accumulation.

(6) $$NUE=\left(\frac{Nt}{Ns}\right)\times \left(\frac{Bw}{Nt}\right)$$

#### 4.2.7. Heterosis Among Different Genotypes

To systematically compare the difference between hybrids and their inbred lines, absolute heterosis (*AH*), mid-parent heterosis (*MPH*), and the range of *MPH* were determined. *MPH*, which measured the superior performance of a hybrid over the mean performance of its inbred lines, was calculated as follows:

(7) $$AH=\left(\frac{F1-BP}{BP}\right)\times 100$$

where *F*1, hybrid performance; *BP*, best parent.

(8) $$MPH=\left(\frac{F1-MP}{MP}\right)\times 100$$

(9) $$MP=\left(\frac{P1+P2}{2}\right)\;$$

where *P*1 and *P*2 are the performance values of the inbred lines, respectively.

#### 4.2.8. Statistical Analysis

Data were statistically analyzed using Statistix and OriginPro 2024b (OriginLab Corporation, Northampton, MA, USA). A two-way ANOVA with a post hoc LSD test was used to compare differences between treatments at a significance level of *p* < 0.05. The figures, principal component analysis, regression equations, coefficient (r), and coefficient of determination (R^2^) were generated using OriginPro 2024b and RStudio software (version 2023.12.1+402). The kinetic parameters were calculated using the Michaelis–Menten equation model, implemented through the “minpack.lm” package in RStudio. Structural equation modeling (SEM) was performed in Rstudio using the Partial Least Squares Path Modeling (PLS-PM) package to test the hypothesized relationship pathways from root traits to NUE. PLS-PM was conducted in Smartpls v2.0 [88].

## 5. Conclusions

This study emphasizes the significant genotypic variability in NUE among maize inbred lines and hybrids under contrasting N conditions. Hybrids consistently outperformed inbred lines, exhibiting superior root traits such as greater activity, length, surface area, and dry weight, all strongly correlated with NUpE. The enzymatic activities of NR and GS in the roots and shoots were strongly associated with NUpE and NUtE, further enhancing NUE in hybrids. Hybrids demonstrated higher N accumulation in the roots, shoots, and total plant biomass, demonstrating their efficient N assimilation and utilization. The hybrid Zheng58 × PH4CV emerged as a top performer, followed by 444 × MO17 and B73 × MO17, due to their robust root architecture, efficient N metabolism, elevated N accumulation, and NUE. These findings provide critical insights for breeding programs, emphasizing the selection of physiological and biochemical traits like enhanced root systems and enzyme activities to develop high-NUE maize hybrids.

## Figures and Tables

**Table 1:** Kinetic parameters of maize genotypes for shoot dry weight (g plant^−1^) under N fertilization.

Genotype	Alpha (α)	Beta (β)	R^2^
Zheng58	5.14 ± 0.280 b	0.022 ± 0.011 b	0.45
Chang 7-2	8.42 ± 0.445 ab	0.289 ± 0.09 ab	0.58
444	9.28 ± 4.049 ab	0.352 ± 0.241 ab	0.68
MO17	8.16 ± 0.500 ab	0.096 ± 0.019 b	0.94
B73	12.85 ± 2.56 a	0.531 ± 0.189 a	0.87
PH6WC	5.06 ± 0.181 b	0.035 ± 0.018 b	0.52
PH4CV	9.93 ± 0.663 ab	0.183 ± 0.031 ab	0.97
Zheng58 × Chang 7-2	15.76 ± 0.887 bc	0.101 ± 0.03 abc	0.75
444 × Chang 7-2	12.68 ± 0.737 cdef	0.067 ± 0.02 bc	0.72
B73 × Chang 7-2	10.21 ± 0.763 f	0.060 ± 0.03 bc	0.62
PH6WC × Chang 7-2	11.23 ± 0.993 f	0.027 ± 0.01 c	0.86
Zheng58 × MO17	11.82 ± 0.382 ef	0.055 ± 0.01 bc	0.82
444 × MO17	17.16 ± 2.092 b	0.106 ± 0.04 ab	0.81
B73 × MO17	13.25 ± 0.568 cdef	0.059 ± 0.01 bc	0.89
PH6WC × MO17	11.96 ± 0.946 def	0.054 ± 0.03 bc	0.71
Zheng58 × PH4CV	22.22 ± 0.274 a	0.170 ± 0.013 a	0.95
444 × PH4CV	15.12 ± 0.359 bcd	0.072 ± 0.01 bc	0.93
B73 × PH4CV	14.96 ± 2.15 bcde	0.085 ± 0.04 bc	0.63
PH6WC × PH4CV	13.48 ± 1.36 cdef	0.074 ± 0.04 bc	0.77

Note: Means followed by the same letters within the same columns are not different statistically at *p* ≥ 0.05; R^2^ represents the coefficient of determination.

**Table 2:** Mean performance of 19 maize genotypes in terms of N accumulation in different plant parts under different N supply.

Genotype	Rank	TNA	RNA	SNA
		Nitrogen supply = 0.153 g/plant		
Zheng 58	15	0.183 ± 0.017	0.044 ± 0.003	0.140 ± 0.015
Chang 7-2	19	0.111 ± 0.022	0.031 ± 0.004	0.081 ± 0.018
444	18	0.121 ± 0.006	0.023 ± 0.001	0.099 ± 0.006
M017	13	0.220 ± 0.003	0.052 ± 0.002	0.168 ± 0.005
B73	16	0.122 ± 0.015	0.035 ± 0.004	0.088 ± 0.014
PH6WC	17	0.172 ± 0.009	0.035 ± 0.002	0.137 ± 0.008
PH4CV	12	0.205 ± 0.013	0.050 ± 0.006	0.155 ± 0.008
Zheng 58 × Chang 7-2	10	0.231 ± 0.023	0.050 ± 0.005	0.182 ± 0.019
444 × Chang 7-2	11	0.219 ± 0.015	0.035 ± 0.004	0.184 ± 0.012
B73 × Chang 7-2	14	0.208 ± 0.016	0.045 ± 0.002	0.163 ± 0.015
PH6WC × Chang 7-2	9	0.264 ± 0.006	0.056 ± 0.005	0.208 ± 0.009
Zheng 58 × MO17	8	0.253 ± 0.007	0.067 ± 0.003	0.186 ± 0.005
444 × MO17	3	0.267 ± 0.015	0.052 ± 0.004	0.214 ± 0.011
B73 × MO17	4	0.295 ± 0.004	0.086 ± 0.006	0.210 ± 0.006
PH6WC × MO17	6	0.284 ± 0.014	0.084 ± 0.002	0.199 ± 0.012
Zheng 58 × PH4CV	1	0.296 ± 0.021	0.069 ± 0.008	0.227 ± 0.014
444 × PH4CV	2	0.292 ± 0.004	0.052 ± 0.001	0.240 ± 0.004
B73 × PH4CV	7	0.264 ± 0.021	0.056 ± 0.004	0.208 ± 0.018
PH6WC × PH4CV	5	0.272 ± 0.014	0.078 ± 0.002	0.194 ± 0.013
Mean	Nitrogen	0.226 ± 0.008	0.052 ± 0.002	0.173 ± 0.006
		Nitrogen supply = 0.46 g/plant		
Zheng 58	15	0.224 ± 0.017	0.056 ± 0.005	0.168 ± 0.016
Chang 7-2	19	0.190 ± 0.019	0.045 ± 0.004	0.145 ± 0.014
444	18	0.206 ± 0.028	0.033 ± 0.003	0.172 ± 0.025
M017	13	0.306 ± 0.013	0.060 ± 0.001	0.246 ± 0.013
B73	16	0.253 ± 0.017	0.046 ± 0.002	0.207 ± 0.015
PH6WC	17	0.181 ± 0.002	0.036 ± 0.002	0.146 ± 0.001
PH4CV	12	0.324 ± 0.010	0.066 ± 0.004	0.258 ± 0.007
Zheng 58 × Chang 7-2	10	0.349 ± 0.007	0.075 ± 0.002	0.275 ± 0.007
444 × Chang 7-2	11	0.317 ± 0.015	0.058 ± 0.003	0.258 ± 0.013
B73 × Chang 7-2	14	0.281 ± 0.010	0.057 ± 0.003	0.224 ± 0.007
PH6WC × Chang 7-2	9	0.317 ± 0.018	0.066 ± 0.006	0.251 ± 0.013
Zheng 58 × MO17	8	0.349 ± 0.008	0.092 ± 0.004	0.257 ± 0.005
444 × MO17	3	0.434 ± 0.022	0.121 ± 0.009	0.313 ± 0.014
B73 × MO17	4	0.395 ± 0.020	0.104 ± 0.007	0.291 ± 0.014
PH6WC × MO17	6	0.358 ± 0.009	0.092 ± 0.010	0.267 ± 0.001
Zheng 58 × PH4CV	1	0.489 ± 0.017	0.110 ± 0.007	0.379 ± 0.013
444 × PH4CV	2	0.441 ± 0.008	0.091 ± 0.002	0.351 ± 0.008
B73 × PH4CV	7	0.376 ± 0.025	0.091 ± 0.011	0.286 ± 0.020
PH6WC × PH4CV	5	0.371 ± 0.013	0.107 ± 0.003	0.263 ± 0.009
Mean	Nitrogen	0.324 ± 0.011	0.073 ± 0.003	0.250 ± 0.008
	ANOVA			
	G	***	***	***
	N	***	***	**
	G × N	***	***	***

Genotypes ranked by total N accumulation (TNA), averaged across both N levels. G, genotype; N, nitrogen; RNA, root N accumulation; SNA, shoot N accumulation; TNA; For ANOVA, ** and *** represent significant differences at *p* ≤ 0.01, and 0.001, respectively. The values are presented as mean ± standard deviation.

**Table 3:** Nitrogen use efficiency and components of N efficiency for 19 genotypes at two levels of N fertilizer.

Genotype	Rank	NUE	NUpE	NutE
		Nitrogen supply = 0.153 g/plant		
Zheng 58	15	42.28 ± 3.06	1.20 ± 0.11	35.44 ± 0.76
Chang 7-2	18	29.80 ± 5.94	0.73 ± 0.14	40.88 ± 0.22
444	19	26.49 ± 1.10	0.79 ± 0.04	33.46 ± 0.46
M017	13	51.28 ± 0.32	1.44 ± 0.02	35.67 ± 0.74
B73	17	29.62 ± 2.92	0.8 ± 0.10	37.26 ± 1.03
PH6WC	16	43.11 ± 0.63	1.19 ± 0.01	36.37 ± 0.18
PH4CV	14	45.09 ± 2.94	1.34 ± 0.09	33.71 ± 0.23
Zheng 58 × Chang 7-2	8	82.39 ± 7.47	1.51 ± 0.15	54.62 ± 0.46
444 × Chang 7-2	11	74.42 ± 5.78	1.43 ± 0.11	51.86 ± 1.08
B73 × Chang 7-2	12	68.39 ± 5.01	1.36 ± 0.11	50.30 ± 0.29
PH6WC × Chang 7-2	9	86.38 ± 1.80	1.72 ± 0.04	50.09 ± 0.73
Zheng 58 × MO17	10	80.72 ± 2.02	1.65 ± 0.05	48.89 ± 1.14
444 × MO17	4	87.19 ± 3.76	1.74 ± 0.09	50.07 ± 0.72
B73 × MO17	3	93.84 ± 0.32	1.93 ± 0.03	49.01 ± 0.64
PH6WC × MO17	5	92.75 ± 5.37	1.85 ± 0.09	50.01 ± 0.78
Zheng 58 × PH4CV	1	94.16 ± 4.88	1.93 ± 0.14	48.80 ± 0.87
444 × PH4CV	2	93.14 ± 1.65	1.91 ± 0.03	48.78 ± 0.43
B73 × PH4CV	7	86.78 ± 6.17	1.73 ± 0.14	50.33 ± 1.12
PH6WC × PH4CV	6	89.83 ± 4.95	1.77 ± 0.09	50.59 ± 0.20
	Nitrogen	68.33 ± 3.34	1.48 ± 0.05	45.06 ± 0.95
		Nitrogen supply = 0.46 g/plant		
Zheng 58	15	15.77 ± 0.45	0.49 ± 0.04	32.59 ± 1.42
Chang 7-2	18	15.88 ± 1.72	0.41 ± 0.04	38.40 ± 0.35
444	19	13.99 ± 1.64	0.45 ± 0.06	31.47 ± 1.42
M017	13	21.32 ± 0.46	0.67 ± 0.03	32.10 ± 0.83
B73	17	17.82 ± 1.17	0.55 ± 0.04	32.43 ± 0.16
PH6WC	16	12.64 ± 0.62	0.37 ± 0.02	33.86 ± 0.46
PH4CV	14	21.8 ± 0.84	0.70 ± 0.02	30.95 ± 0.38
Zheng 58 × Chang 7-2	8	37.46 ± 1.32	0.76 ± 0.02	49.33 ± 0.96
444 × Chang 7-2	11	31.72 ± 1.45	0.69 ± 0.03	46.10 ± 0.59
B73 × Chang 7-2	12	26.97 ± 1.35	0.61 ± 0.02	44.12 ± 0.81
PH6WC × Chang 7-2	9	31.44 ± 1.68	0.69 ± 0.04	45.68 ± 0.51
Zheng 58 × MO17	10	32.84 ± 1.07	0.76 ± 0.02	43.31 ± 0.46
444 × MO17	4	43.90 ± 2.80	0.94 ± 0.05	46.48 ± 0.65
B73 × MO17	3	37.07 ± 1.56	0.86 ± 0.04	43.16 ± 0.32
PH6WC × MO17	5	34.32 ± 1.51	0.78 ± 0.02	44.00 ± 0.93
Zheng 58 × PH4CV	1	47.16 ± 1.21	1.06 ± 0.04	44.35 ± 0.52
444 × PH4CV	2	40.38 ± 0.88	0.96 ± 0.02	42.09 ± 0.18
B73 × PH4CV	7	38.34 ± 3.06	0.82 ± 0.06	46.87 ± 1.49
PH6WC × PH4CV	6	36.88 ± 0.85	0.81 ± 0.03	45.82 ± 0.72
	Nitrogen	29.35 ± 1.43	0.7 ± 0.02	40.69 ± 0.83
	ANOVA			
	G	***	***	***
	N	***	***	**
	G × N	***	***	*

Genotypes ranked by NUE averaged across both N levels. G, genotype; N, nitrogen; NUE, N use efficiency; NUpE, N uptake efficiency; NUtE, N utilization efficiency; For ANOVA, *, **, and *** represent significant differences at *p* ≤ 0.05, 0.01, and 0.001, respectively. The values are presented as mean ± standard deviation.

**Table 4:** Absolute heterosis (AH) value, mid-parental heterosis (MPH) value, and range of mid-parental heterosis for different parameters of maize genotypes.

Variable	Unit	AH (%)	MPH (%)	Range of MPH (%)
Root activity	µg/g/h	42.74	58.94	33.03 to 89.70
Root length	cm	42.78	61.03	4.88 to 116.08
Root surface area	cm^2^	15.39	34.10	−0.82 to 85.24
Root volume	cm^3^	32.71	66.02	−9.95 to 217.30
Root diameter	Mm	6.43	11.70	−5.58 to 40.75
Root NR activity	nmol/g/h FW	−2.56	4.44	−14.07 to 18.82
Root GS activity	U/g FW	8.40	12.57	0.04 to 30.52
Leaf NR activity	nmol/g/h FW	56.45	60.91	53.07 to 68.04
Leaf GS activity	U/g FW	15.31	26.18	7.88 to 43.69
Free amino acid	mg/g FW	−27.13	−19.83	−44.71 to −0.63
Nitrate content	mg/g FW	4.97	8.92	−1.24 to 20.25
soluble protein	mg/g FW	1.69	4.54	−1.58 to 16.35
Soluble sugar	mg/g FW	−18.54	−13.17	−31.10 to 1.88
Chlorophyll a	mg/g FW	3.72	11.77	−8.12 to 36.70
Chlorophyll b	mg/g FW	−1.82	10.52	−10.88 to 49.03
Chlorophyll a+b	mg/g FW	3.56	11.35	−4.11 to 37.16
Carotenoid	mg/g FW	2.56	12.17	−17.28 to 49.07
Photosynthetic rate	µmol m^−2^ s^−1^	0.16	3.56	−12.12 to 25.87
Transpiration rate	mmol m^−2^ s^−1^	0.33	3.63	−7.71 to 12.65
Stomatal conductance	mmol H_2_O m^−2^ s^−1^	3.80	9.81	−10.30 to 25.04
Intercellular CO_2_ concentration	µmol CO_2_ mol^−1^ air	3.36	5.76	−3.91 to 14.61
Fv/Fm	-	8.97	11.52	5.04 to 28.17
Y (II)	-	6.60	10.91	−0.55 to 22.33
Leaf area	cm^2^/plant	43.94	56.48	25.39 to 84.23
Plant height	m	25.40	32.15	20.62 to 48.78
Root dry weight	g/plant	72.92	102.20	60.77 to 137.44
Shoot dry weight	g/plant	100.79	122.10	81.96 to 154.16
Total dry weight	g/plant	92.01	116.05	75.01 to 139.47
Root N concentration	%	−21.03	−18.05	−30.72 to −3.99
Shoot N concentration	%	−34.31	−30.56	−37.29 to −21.97
Total N concentration	%	−28.0	−25.89	−29.59 to −21.98
Root N accumulation	g/plant	43.98	66.79	28.83 to 105.76
Shoot N accumulation	g/plant	32.90	53.65	22.72 to 80.49
Total N accumulation	g/plant	35.99	56.29	28.84 to 77.42
NUpE	g/g	37.09	58.19	27.19 to 78.77
NutE	g/g	32.69	37.65	26.76 to 45.51
NUE	g/g	94.30	119.81	73.83 to 148.68

FW, fresh weight.

**Table 5:** Maize inbred lines: breeding origins, release timeline, and their hybrids.

Inbred Lines	Breeding/Provide Institution	Year
Zheng 58	Henan Academy of Agricultural Sciences	1993
Chang 7-2	Henan Academy of Agricultural Sciences	1991
444	Jilin Academy of Agricultural Sciences	1984
MO17	University of Missouri, USA	1970s
B73	Lowa State University, USA	1972
PH6WC	Pioneer Hi-Bred, USA	1997
PH4CV	Pioneer Hi-Bred, USA	1996
**Hybrids**		
Zheng58 × Chang7-2	Zheng58 × MO17	Zheng58 × PH4CV
444 × Chang7-2	444 × MO17	444 × PH4CV
B73 × Chang7-2	B73 × MO17	B73 × PH4CV
PH6WC × Chang7-2	PH6WC × MO17	PH6WC × PH4CV

## Data Availability

The original contributions presented in the study are included in the article. Further inquiries can be directed to the corresponding author(s).

## Supplementary Materials

The following supporting information can be downloaded at: https://www.mdpi.com/article/10.3390/plants14030399/s1, Figure S1: Response of root activity in maize genotypes to different nitrogen levels at V5 and V7 growth stages; Figure S2: Primary root characteristics of maize genotypes at V7 stage under varying nitrogen supply; Figure S3: Genotypic variation in metabolites at V7 growth stage under varying nitrogen supply; Figure S4: Variation in physiological traits of maize genotypes at V7 growth stage under different nitrogen conditions; Figure S5: Genotypic variation in photosynthetic performance and chlorophyll fluorescence of maize under different nitrogen supply at V5 and V7 growth stages; Figure S6: Genotypic variation in growth traits of maize at V5 and V7 stages under different nitrogen supply; Figure S7: Genotypic variation in plant nitrogen concentration at V7 stage under different nitrogen supply; Table S1: Regression equations and correlation coefficients between total dry matter (TDW) and growth, physiological, and biochemical parameters of maize genotypes under high and low nitrogen supply; Table S2: Regression equations and correlation coefficients between N uptake efficiency (NUpE) and growth, physiological, and biochemical parameters of maize genotypes under high and low nitrogen supply; Table S3: Regression equations and correlation coefficients between N utilization efficiency (NUtE) and growth, physiological, and biochemical parameters of maize genotypes under high and low nitrogen supply.
