# Supplementary material for: Multivariate Analysis of Root Architecture, Morpho-Physiological, and Biochemical Traits Reveals Higher Nitrogen Use Efficiency Heterosis in Maize Hybrids During Early Vegetative Growth
Source: Plants (Basel). 2025 Jan 29;14(3):399. doi: 10.3390/plants14030399 (PMC11821247; doi:10.3390/plants14030399)
Supplement: Supplementary file 1 [file plants-14-00399-s001.zip › plants-3391634-Supplementary Figures.pdf]

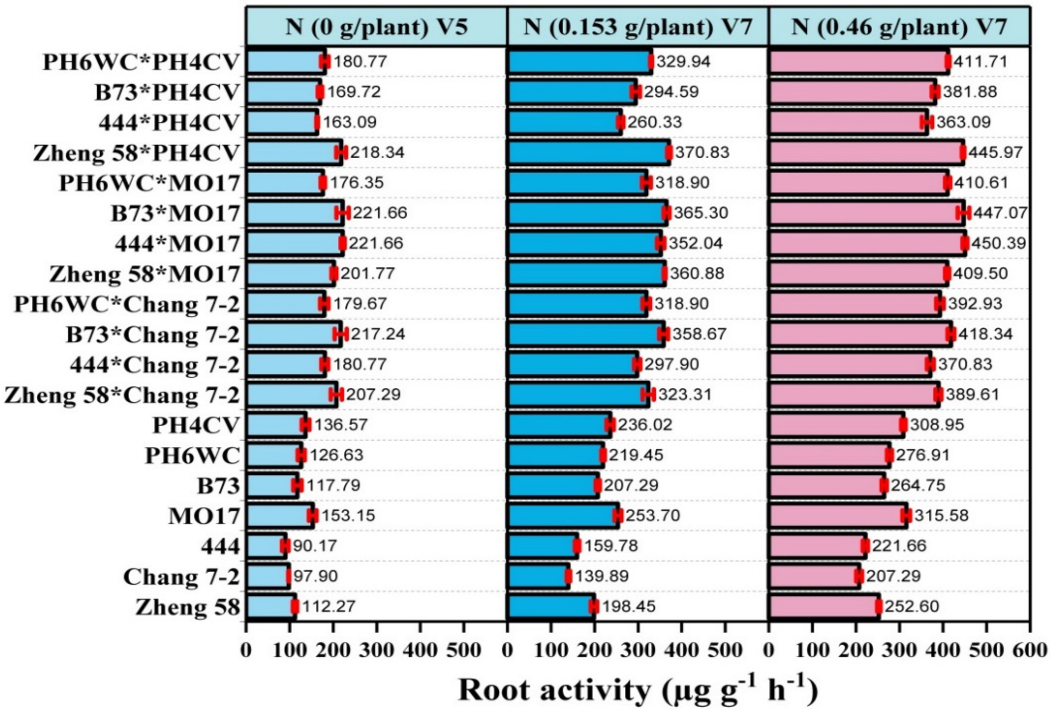

Supplementary Figure S1. Response of root activity in maize genotypes to different nitrogen levels at V5 and V7 growth stages.

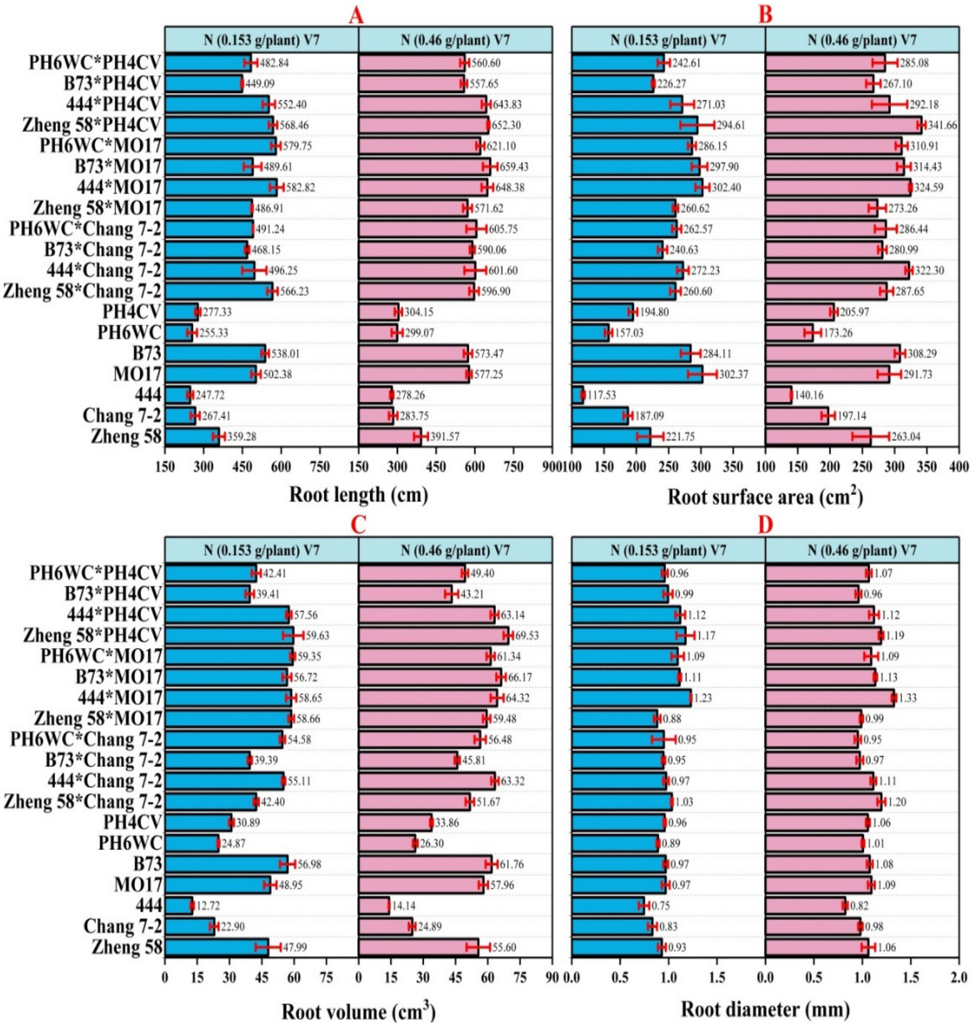

Supplementary Figure S2. Primary root characteristics of maize genotypes at V7 stage under varying nitrogen supply: (A) Root length, (B) Root surface area, (C) Root volume, and (D) Root diameter.

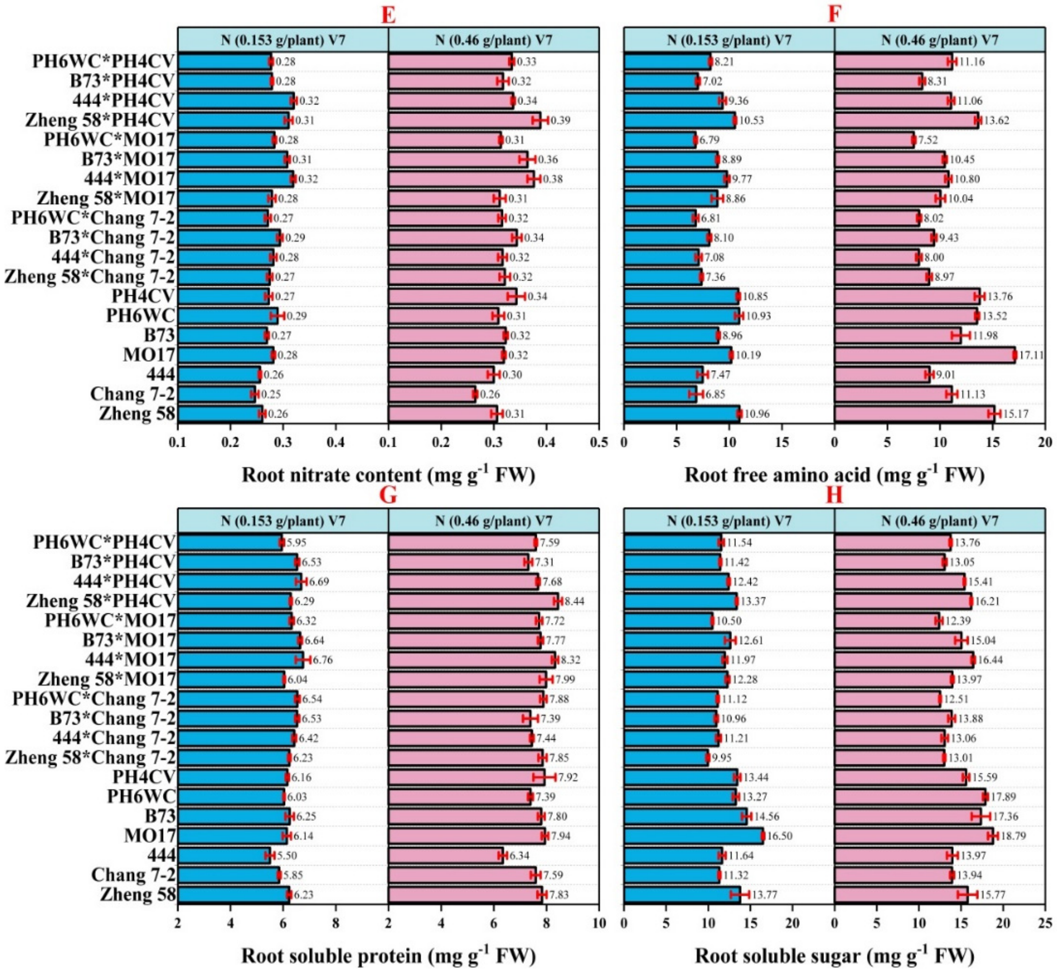

Supplementary Figure S3. Genotypic variation in metabolites at V7 growth stage under varying nitrogen Supply: (E) Nitrate content, (F) Free amino acids, (G) Soluble protein, and (H) Soluble sugar.

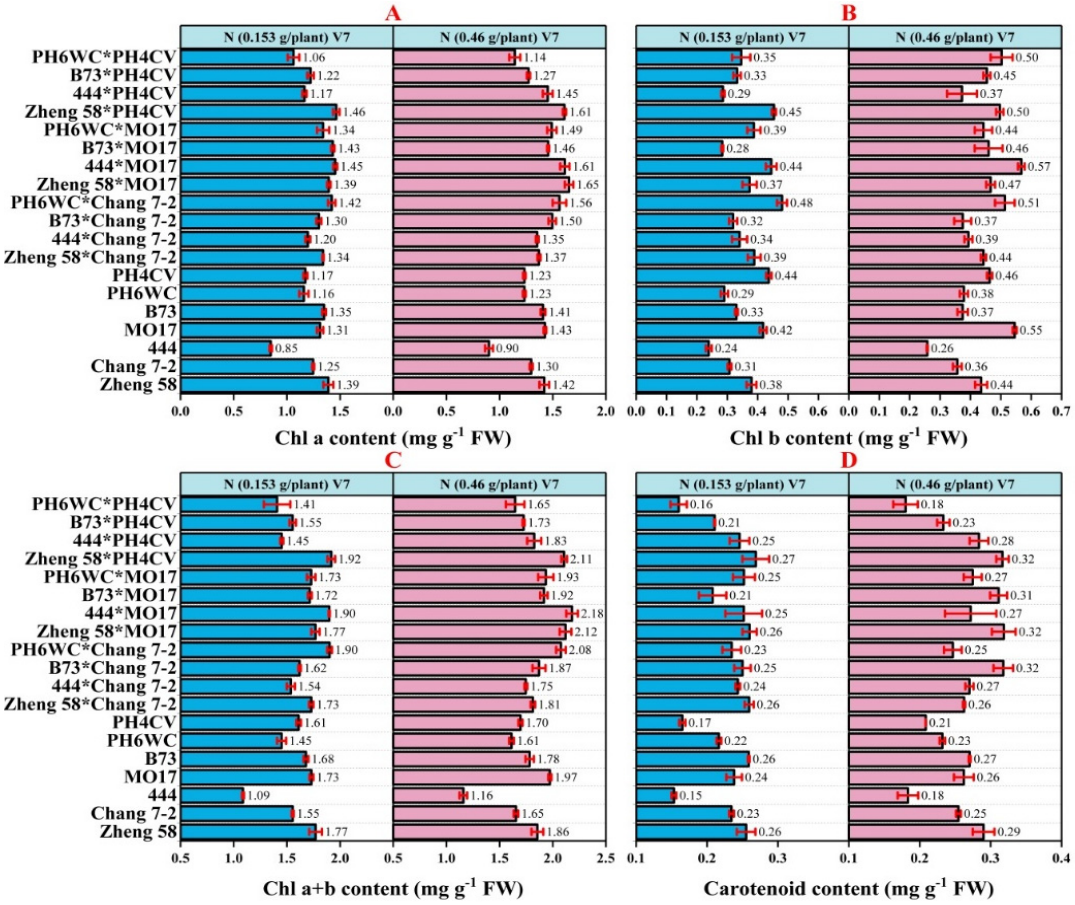

Supplementary Figure S4. Variation in physiological traits of maize genotypes at V7 growth stage under different nitrogen conditions: (A) Chlorophyll a, (B) Chlorophyll b, (C) Total chlorophyll (a + b), and (D) Carotenoid content.

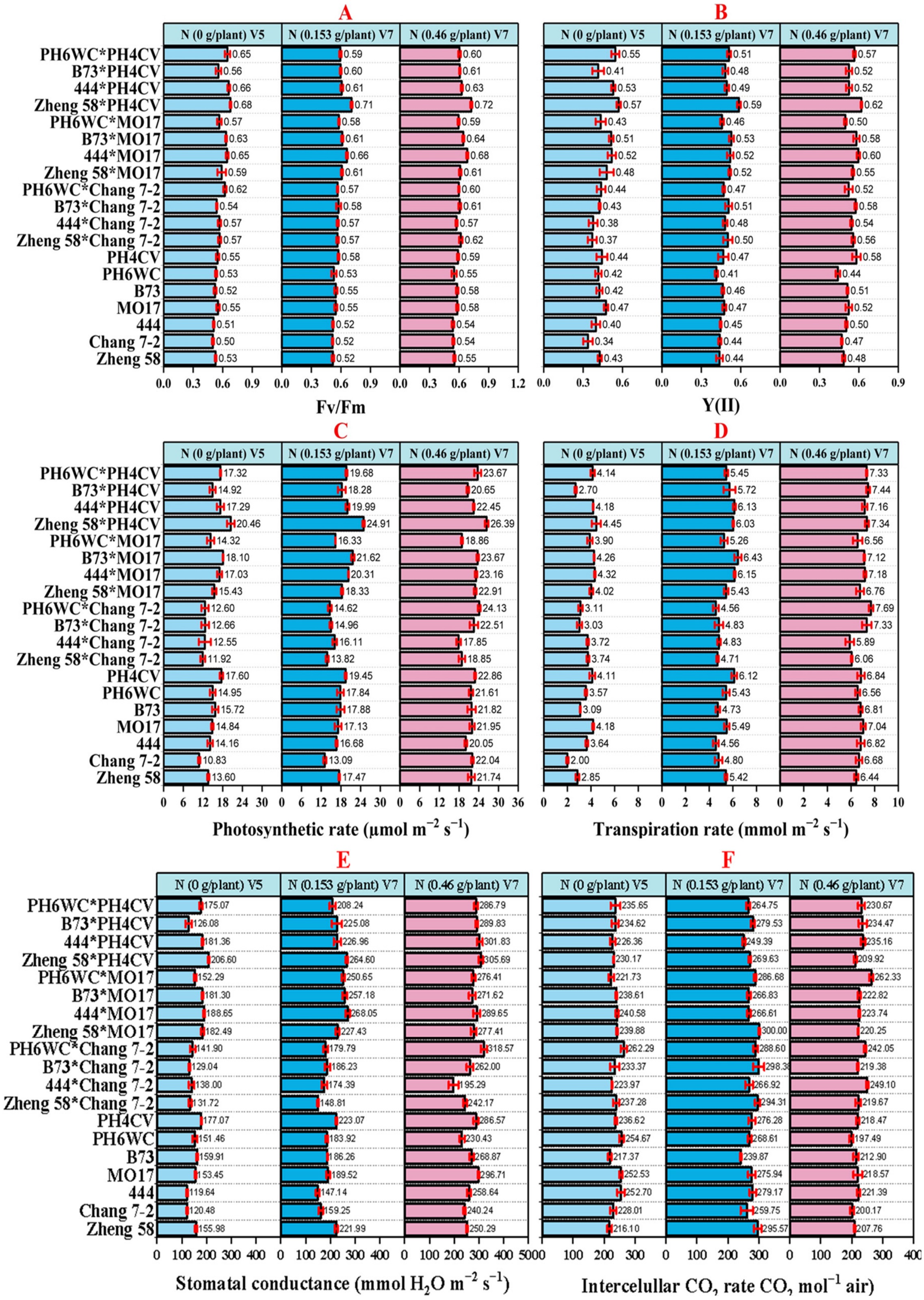
A
B
C
D
E
F
N (0 g/plant) V5
N (0.153 g/plant) V7
N (0.46 g/plant) V7
PH6WC*PH4CV
B73*PH4CV
444*PH4CV
Zheng 58*PH4CV
PH6WC*MO17
B73*MO17
444*MO17
Zheng 58*MO17
PH6WC*Chang 7-2
B73*Chang 7-2
444*Chang 7-2
Zheng 58*Chang 7-2
PH4CV
PH6WC
B73
MO17
444
Chang 7-2
Zheng 58
Fv/Fm
Y(II)
Photosynthetic rate (μmol m^{-2} s^{-1})
Transpiration rate (mmol m^{-2} s^{-1})
Stomatal conductance (mmol H_2O m^{-2} s^{-1})
Intercelullar CO, rate CO, mol^{-1} air)

Supplementary Figure S5. Genotypic variation in photosynthetic performance and chlorophyll fluorescence of maize under different nitrogen supply at V5 and V7 growth stages: (A) Fv/Fm, (B) Y(II), (C) Photosynthetic rate, (D) Transpiration rate, (E) Stomatal conductance, and (F) Intercellular CO_2 concentration rate.

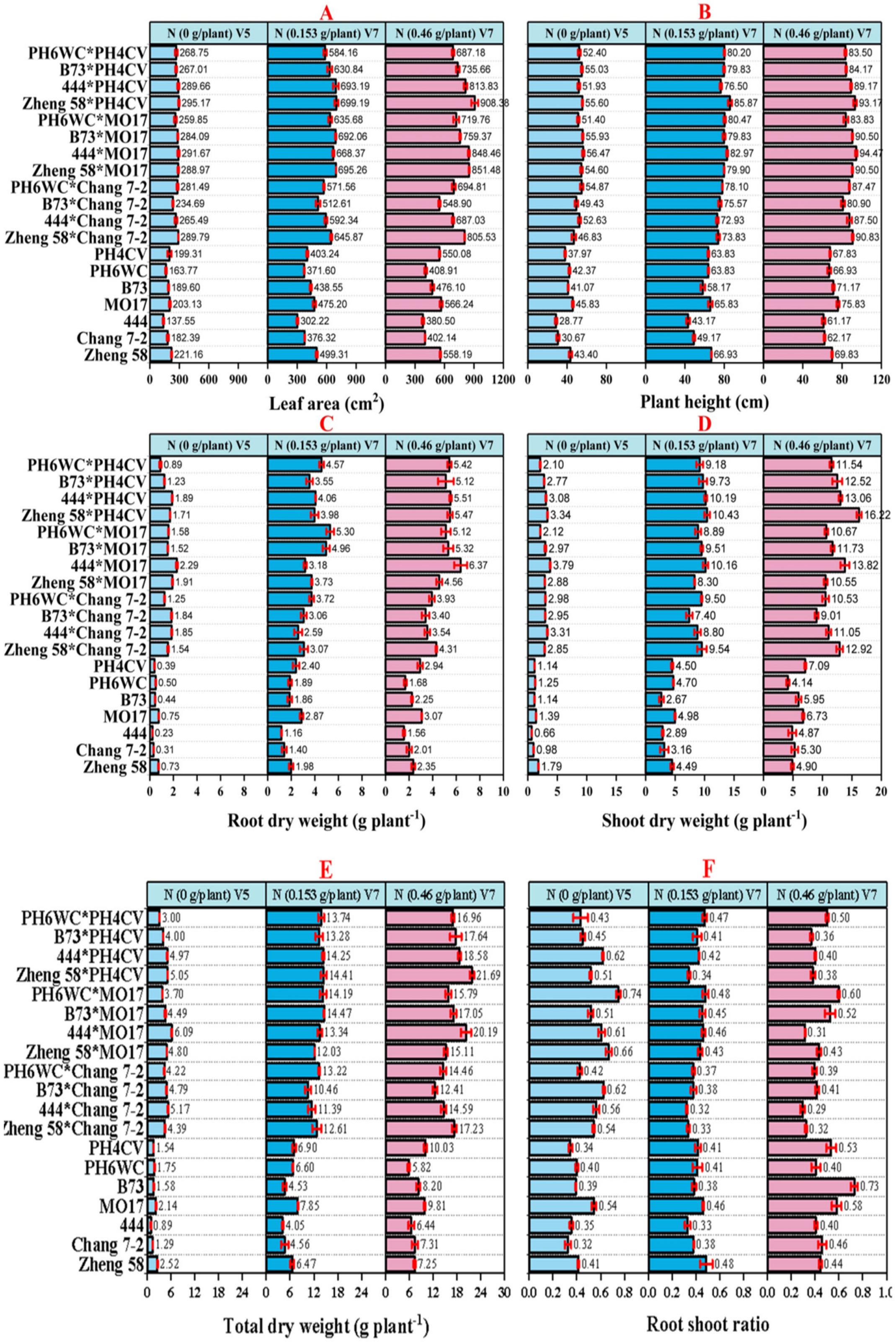

Supplementary Figure S6. Genotypic variation in growth traits of maize at V5 and V7 stages under different nitrogen supply: (A) Leaf area per plant, (B) Plant height, (C) Root dry weight, (D) Shoot dry weight, (E) Total dry matter per plant, and (F) Root-to-shoot ratio

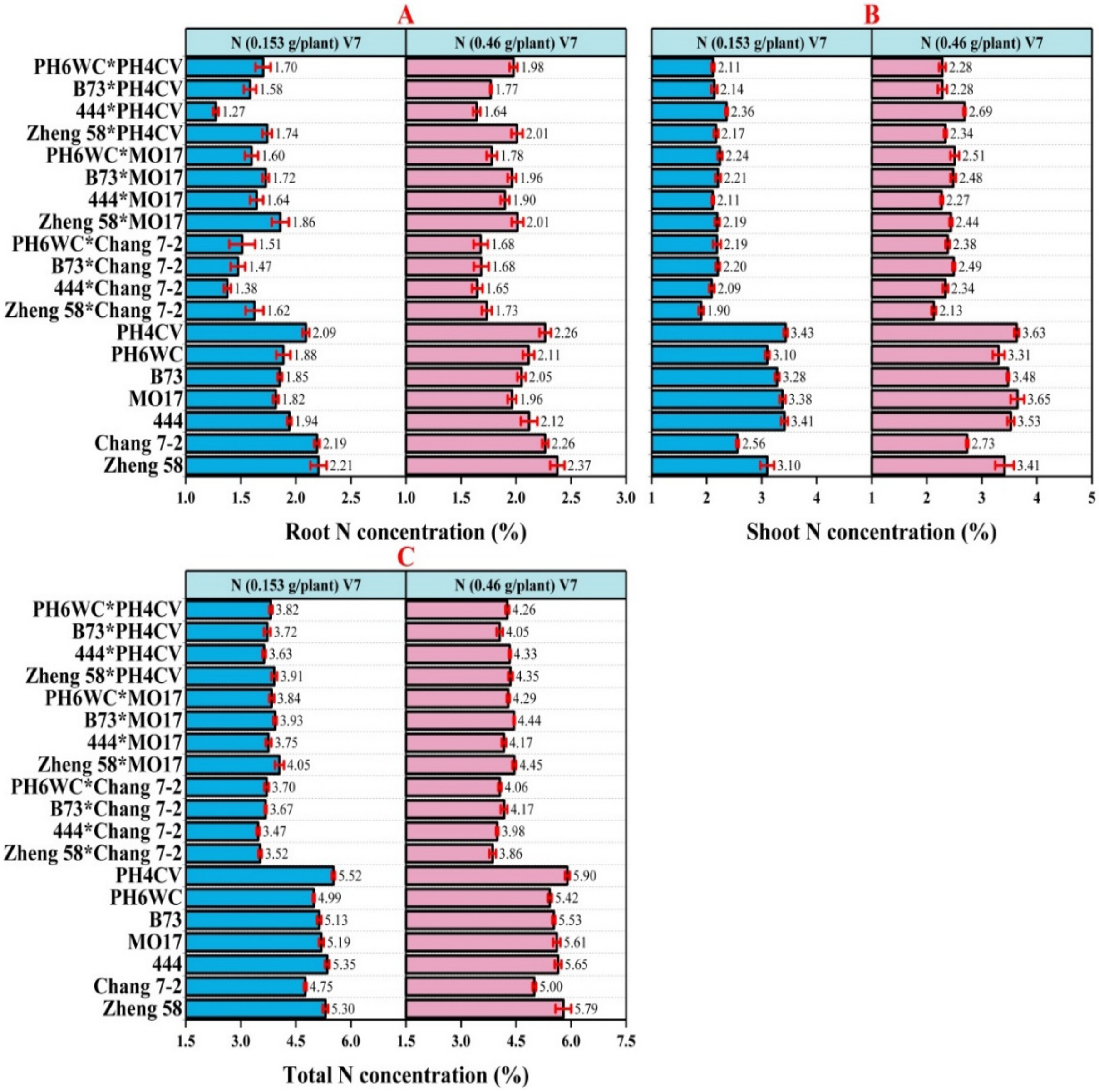
A
N (0.153 g/plant) V7
N (0.46 g/plant) V7
PH6WC*PH4CV
B73*PH4CV
444*PH4CV
Zheng 58*PH4CV
PH6WC*MO17
B73*MO17
444*MO17
Zheng 58*MO17
PH6WC*Chang 7-2
B73*Chang 7-2
444*Chang 7-2
Zheng 58*Chang 7-2
PH4CV
PH6WC
B73
MO17
444
Chang 7-2
Zheng 58
1.70
1.58
1.27
1.74
1.60
1.72
1.64
1.86
1.51
1.47
1.38
1.62
2.09
1.88
1.85
1.82
1.94
2.19
2.21
1.98
1.77
1.64
2.01
1.78
1.96
1.90
2.01
1.68
1.68
1.65
1.73
2.26
2.11
2.05
1.96
2.12
2.26
2.37
1.0 1.5 2.0 2.5 1.0 1.5 2.0 2.5 3.0
Root N concentration (%)
B
N (0.153 g/plant) V7
N (0.46 g/plant) V7
2.11
2.14
2.36
2.17
2.24
2.21
2.11
2.19
2.19
2.20
2.09
1.90
3.43
3.10
3.28
3.38
3.41
2.56
3.10
2.28
2.28
2.69
2.34
2.51
2.48
2.27
2.44
2.38
2.49
2.34
2.13
3.63
3.31
3.48
3.65
3.53
2.73
3.41
1 2 3 4 1 2 3 4 5
Shoot N concentration (%)
C
N (0.153 g/plant) V7
N (0.46 g/plant) V7
PH6WC*PH4CV
B73*PH4CV
444*PH4CV
Zheng 58*PH4CV
PH6WC*MO17
B73*MO17
444*MO17
Zheng 58*MO17
PH6WC*Chang 7-2
B73*Chang 7-2
444*Chang 7-2
Zheng 58*Chang 7-2
PH4CV
PH6WC
B73
MO17
444
Chang 7-2
Zheng 58
3.82
3.72
3.63
3.91
3.84
3.93
3.75
4.05
3.70
3.67
3.47
3.52
5.52
4.99
5.13
5.19
5.35
4.75
5.30
4.26
4.05
4.33
4.35
4.29
4.44
4.17
4.45
4.06
4.17
3.98
3.86
5.90
5.42
5.53
5.61
5.65
5.00
5.79
1.5 3.0 4.5 6.0 1.5 3.0 4.5 6.0 7.5
Total N concentration (%)

Supplementary Figure S7. Genotypic variation in plant nitrogen concentration at V7 stage under different nitrogen Supply: (A) Root N concentration, (B) Shoot N concentration, and (C) Total plant N concentration
