# Supplementary material for: Multivariate Analysis of Root Architecture, Morpho-Physiological, and Biochemical Traits Reveals Higher Nitrogen Use Efficiency Heterosis in Maize Hybrids During Early Vegetative Growth
Source: Plants (Basel). 2025 Jan 29;14(3):399. doi: 10.3390/plants14030399 (PMC11821247; doi:10.3390/plants14030399)
Supplement: Supplementary file 1 [file plants-14-00399-s001.zip › plants-3391634-supplementary tables.pdf]

Supplementary Table S1. Regression equations and correlation coefficients between total dry matter (TDW) and growth, physiological, and biochemical parameters of maize genotypes under high and low nitrogen supply.

		**High N**			**Low N**		
**Variable *Y***	**Variable *X***	**Regression equation**	**R^2**	**Coefficient (*r*)**	**Regression equation**	**R^2**	**Coefficient (*r*)**
TDM	Root activity	Y=13.512x+172.28	0.75***	0.86***	Y=15.639x+118.65	0.71*	0.84***
	Root length	Y=21.647x+234.91	0.59	0.77***	Y=21.536x+231.33	0.49	0.70***
	Root surface area	Y=7.8024x+166.56	0.46	0.68***	Y=8.1047x+161.76	0.29	0.54***
	Root volume	Y=2.0361x+23.477	0.41	0.64***	Y=2.3136x+21.591	0.38*	0.62***
	Root diameter	Y=0.0123x+0.08981	0.26	0.51***	Y=0.0206x+0.7752	0.35*	0.59***
	Root dry matter	Y=0.2925x+0.0588	0.91**	0.95***	Y=0.279x+0.2041	0.81*	0.90***
	RNR activity	Y=6.178x+321.54	0.40**	0.63***	Y=0.7299x+275.02	0.01	0.06
	RGS activity	Y=0.6865x+26.513	0.67***	0.82***	Y=0.3927x+26.487	0.25***	0.51***
	SNR activity	Y=22.031x+255.11	0.82***	0.91***	Y=16.139x+143.27	0.89***	0.94***
	SGS activity	Y=1.2714x+36.791	0.46***	0.68***	Y=1.4831x+31.658	0.44	0.66***
	Root nitrate content	Y=0.0038x+0.2747	0.37***	0.61***	Y=0.0033x+0.2481	0.35***	0.59***
	Root soluble protein	Y=0.0445x+7.0937	0.20***	0.44***	Y=0.0541x+5.7076	0.35***	0.59***
	Root-free amino acid	Y=0.1789x+13.419	0.11*	-0.33	Y=-0.0711x+9.421	0.03	-0.18
	Root soluble sugar	Y=-0.1091x+16.317	0.07*	-0.27	Y=-0.1646x+14.018	0.15**	-0.38
	Chl a+b	Y=0.0268x+1.4639	0.31***	0.56***	Y=0.0194x+1.4369	0.13**	0.36**
	Fv/Fm	Y=0.0078x+0.4962	0.64***	0.80***	Y=0.0092x+0.4828	0.48***	0.69***
	Y(II)	Y=0.0065x+0.4478	0.40***	0.63***	Y=0.007x+0.4111	0.32***	0.57***
	Photosynthetic rate	Y=0.0972x+20.644	0.05	0.21	Y=0.2563x+15.083	0.11*	0.33*
	Leaf area	Y=29.896x+249.1	0.84***	0.91***	Y=28.751x+250.88	0.78***	0.88***
	Plant height	Y=1.972x+53.949	0.81***	0.90***	Y=2.6822x+43.391	0.80***	0.89***
	Shoot dry matter	Y=0.7075x+0.0588	0.98***	0.99***	Y=0.721x+0.2041	0.96	0.98***
	Total N concentration	Y=-0.1118x+6.2097	0.63***	-0.79***	Y=0.1594x+5.9387	0.72***	-0.84***
	Root N accumulation	Y=0.0049x+0.0078	0.81***	0.90***	Y=0.0037x+0.0144	0.59***	0.77***
	Shoot N accumulation	Y=0.0121x+0.087	0.84***	0.91***	Y=0.0116x+0.0517	0.87***	0.93***
	Total N accumulation	Y=0.017x+0.0948	0.90***	0.95***	Y=0.0153x+0.0661	0.91***	0.95***
	NUpE	Y=0.0369x+0.206	0.90***	0.95***	Y=0.0998x+0.4318	0.91***	0.95***
	NutE	Y=1.0282x+26807	0.66***	0.81***	Y=1.5657x+28.69	0.70***	0.84***

R^2, Coefficient of determination; r, correlation; * p <0.05, ** p<0.01, *** p<0.001

Supplementary Table S2. Regression equations and correlation coefficients between N uptake efficiency (NUpE) and growth, physiological, and biochemical parameters of maize genotypes under high and low nitrogen supply.

		**High N**			**Low N**		
**Variable *Y***	**Variable *X***	**Regression equation**	**R^2**	**Coefficient (*r*)**	**Regression equation**	**R^2**	**Coefficient (*r*)**
NUpE	Root activity	Y=319.78x+129.65	0.63***	0.80***	Y=138.58x+77.88	0.62***	0.78***
	Root length	Y=526.48x+156.65	0.53***	0.73***	Y=181.43x+188.78	0.39***	0.62***
	Root surface area	Y=196.77x+133.41	0.44***	0.67***	Y=73.706x+137.74	0.26***	0.52***
	Root volume	Y=54.328x+12.731	0.44***	0.66***	Y=21.4x+14.205	0.36***	0.60***
	Root diameter	Y=0.3214x+0.8378	0.27***	0.52***	Y=0.1934x+0.7048	0.34***	0.58***
	Root dry matter	Y=7.2461-1.2091	0.85***	0.92***	Y=2.6725x-0.8219	0.82***	0.91***
	RNR activity	Y=175.24x+281.62	0.48***	0.70***	Y=15.408x+259.92	0.02	0.15
	RGS activity	Y=18.223x+22.956	0.71***	0.84***	Y=4.0886x+24.56	0.31***	0.55***
	SNR activity	Y=489.5x+208.06	0.61***	0.78***	Y=136.7x+110.3	0.70***	0.84***
	SGS activity	Y=33.168x+30.614	0.47***	0.69***	Y=13.603x+27.091	0.41***	0.64***
	Root nitrate content	Y=0.1131x+0.2468	0.49***	0.70***	Y=0.0324x+0.2347	0.37***	0.61***
	Root soluble protein	Y=1.3323x+6.7576	0.27***	0.52***	Y=0.5069x+5.5248	0.34***	0.58***
	Root free amino acid	Y=-1.8333x+12.294	0.02	-0.13	Y=0.1369x+8.4755	0.001	0.04
	Root soluble sugar	Y=-1.0101x+15.555	0.01	-0.09	Y=-0.8142x+13.498	0.04	-0.21
	Chl a+b	Y=0.6938x+1.3379	0.31***	0.56***	Y=0.1878x+1.3619	0.14***	0.37***
	Fv/Fm	Y=0.2057x+0.4565	0.67***	0.82***	Y=0.0859x+0.4516	0.46***	0.68***
	Y(II)	Y=0.1789x+0.4097	0.46***	0.68***	Y=0.063x+0.3915	0.28***	0.53***
	Photosynthetic rate	Y=3.5536x+19.455	0.09*	0.31*	Y=3.3338x+12.844	0.21***	0.46***
	Leaf area	Y=741.69x+130.74	0.78***	0.88***	Y=251.28x+180.71	0.65***	0.81***
	Plant height	Y=46.505x+47.844	0.68***	0.83***	Y=24.688x+35.006	0.74***	0.86***
	Shoot dry matter	Y=17.158x-2.4651	0.87***	0.94***	Y=6.4089x-21.1227	0.84***	0.92***
	Total dry matter	Y=24.404-3.6743	0.90***	0.95***	Y=9.0813x-2.9446	0.91***	0.95***
	Total N concentration	Y=-2.1009x+6.1786	0.33***	-0.58***	Y=-1.1825x+6.0168	0.43***	-0.66***
	Root N accumulation	Y=0.1259x-0.0147	0.81***	0.90***	Y=0.0371x-0.0022	0.67***	0.82***
	Shoot N accumulation	Y=0.3341x+0.0147	0.97***	0.98***	Y=0.1159x+0.0022	0.95***	0.97***
	Total N accumulation	Y=0.46x-0.00	1***	1***	Y=0.153x-0.00	1***	1***
	NutE	Y=19.412x+27.027	0.36***	0.60***	Y=11.411x+28.222	0.41***	0.64***

R^2, Coefficient of determination; r, correlation; * *p* <0.05, ***p*<0.01, ****p*<0.001

Supplementary Table S3. Regression equations and correlation coefficients between N utilization efficiency (NUtE) and growth, physiological, and biochemical parameters of maize genotypes under high and low nitrogen supply.

		**High N**			**Low N**		
**Variable *Y***	**Variable *X***	**Regression equation**	**R^2**	**Coefficient (*r*)**	**Regression equation**	**R^2**	**Coefficient (*r*)**
NutE	Root activity	Y=9.6682x-38.672	0.61***	0.78***	Y=7.7258x-65.76	0.61***	0.78***
	Root length	Y=14.84x-76.63	0.44***	0.67***	Y=11.196x-47.99	0.46***	0.68***
	Root surface area	Y=4.9862x+69.022	0.30***	0.55***	Y=3.9052x+70.526	0.24***	0.48***
	Root volume	Y=1.132x+4.9093	0.20***	0.45***	Y=1.0431-1.2217	0.27***	0.52***
	Root diameter	Y=0.0064x+0.8042	0.11*	0.33*	Y=0.0089x+0.5881	0.23***	0.48***
	Root dry matter	Y=0.1817x-3.5008	0.56***	0.75***	Y=0.1103x-1.8483	0.44***	0.67***
	RNR activity	Y=2.7096x+294.71	0.12***	0.35***	Y=-0.4399x+302.47	0.01	-0.08
	RGS activity	Y=0.3514x+21.482	0.28***	0.53***	Y=0.1257x+24.93	0.09*	0.30*
	SNR activity	Y=17.467x-158.14	0.82***	0.90***	Y=8.3074x-62.323	0.79***	0.86***
	SGS activity	Y=0.6973x+25.059	0.31***	0.56***	Y=0.704x+25.059	0.34***	0.59***
	Root nitrate content	Y=0.0013x+0.2736	0.07*	0.26*	Y=0.0013x+0.2261	0.17**	0.42**
	Root soluble protein	Y=0.0167x+7.0164	0.04	0.21	Y=0.0243x+5.1772	0.25***	0.50***
	Root-free amino acid	Y=-0.2673x+21.88	0.40***	-0.63***	Y=-0.1028x+21.88	0.22***	-0.47***
	Root soluble sugar	Y=-0.1768x+22.039	0.32***	-0.56***	Y=-0.1388x+18.55	0.37***	-0.61***
	Chl a+b	Y=0.0164x+1.1586	0.19***	0.43***	Y=0.0094x+1.2165	0.11*	0.33*
	Fv/Fm	Y=0.004x+0.4379	0.27***	0.52***	Y=0.0038x+0.408	0.29***	0.53***
	Y(II)	Y=0.0032x+0.4049	0.16***	0.40***	Y=0.0033x+0.3378	0.24***	0.49***
	Photosynthetic rate	Y=0.0333x+20.589	0.01	0.10	Y=-0.0315x+19.047	0.01	0.08
	Leaf area	Y=18.875x-115.23	0.53***	0.73***	Y=14.219x-89.211	0.66***	0.81***
	Plant height	Y=1.4038x+23.454	0.66***	0.81***	Y=1.2338x+15.84	0.59***	0.77***
	Shoot dry matter	Y=0.4633x-9.2412	0.67***	0.82***	Y=0.3397x-7.9726	0.75***	0.86***
	Total dry matter	Y=0.645x-12.742	0.66***	0.81***	Y=0.45x-9.8209	0.70***	0.84***
	Total N concentration	Y=-0.1102x+9.1825	0.97***	-0.99***	Y=-0.0994x+8.7499	0.97***	-0.98***
	Root N accumulation	Y=0.0027x-0.0377	0.41***	0.64***	Y=0.0012x-0.0036	0.24***	0.49***
	Shoot N accumulation	Y=0.0057x+0.0171	0.30***	0.55***	Y=0.0043x-0.0192	0.41***	0.64***
	Total N accumulation	Y=0.0085x-0.0206	0.36***	0.60***	Y=0.0055x-0.0228	0.41***	0.64***
	NUpE	Y=0.0184x-0.0448	0.36***	0.60***	Y=0.0361x-0.0448	0.41***	0.64***

R^2, Coefficient of determination; r, correlation; * p <0.05, **p<0.01, ***p<0.001
